# Stacking Tolerance to Drought and Resistance to a Parasitic Weed in Tropical Hybrid Maize for Enhancing Resilience to Stress Combinations

**DOI:** 10.3389/fpls.2020.00166

**Published:** 2020-02-28

**Authors:** Abebe Menkir, José Crossa, Silvestro Meseka, Bunmi Bossey, Oyekunle Muhyideen, Priscillia F. Riberio, Mmadou Coulibaly, Abdoul-Madjidou Yacoubou, Gbadebo Olaoye, Alidu Haruna

**Affiliations:** ^1^ International Institute of Tropical Agriculture (IITA), Ibadan, Nigeria; ^2^ Biometrics and Statistics Unit, International Maize and Wheat Improvement Center (CIMMYT), Mexico City, Mexico; ^3^ Department of Plant Science, Institute for Agricultural Research/Ahmadu Bello University, Zaria, Nigeria; ^4^ Cereals Division, CSIR-Crops Research Institute, Kumasi, Ghana; ^5^ Maize Improvement Program, Institute de Economic Rurale, Bamako, Mali; ^6^ Crop Breeding Department, National Institute of Agricultural Research of Benin/CRA, Cotonou, Benin; ^7^ Department of Agronomy, University of Ilorin, Ilorin, Nigeria; ^8^ Maize Improvement Program, CSIR-Savanna Agricultural Research Institute, Tamale, Ghana

**Keywords:** stress combination, tolerance to drought, resistance to *Striga hermonthica*, Managed drought stress, artificial infestation, multi-environment trial

## Abstract

Maize is a food security crop cultivated in the African savannas that are vulnerable to the occurrence of drought stress and *Striga hermonthica* infestation. The co-occurrence of these stresses can severely damage crop growth and productivity of maize. Until recently, maize breeding in International Institute of Tropical Agriculture (IITA) has focused on the development of either drought tolerant or *S. hermonthica* resistant germplasm using independent screening protocols. The present study was therefore conducted to examine the extent to which maize hybrids simultaneously expressing resistance to *S. hermonthica* and tolerance to drought (DTSTR) could be developed through sequential selection of parental lines using the two screening protocols. Regional trials involving 77 DTSTR and 22 commercial benchmark hybrids (STR and non-DTSTR) were then conducted under *Striga-*infested and non-infested conditions, managed drought stress and fully irrigated conditions as well as in multiple rainfed environments for 5 years. The observed yield reductions of 61% under managed drought stress and 23% under *Striga-*infestation created desirable stress levels leading to the detection of significant differences in grain yield among hybrids at individual stress and non-stress conditions. On average, the DTSTR hybrids out-yielded the STR and non-DTSTR commercial hybrids by 13–19% under managed drought stress and fully irrigated conditions and by −4 to 70% under *Striga-*infested and non-infested conditions. Among the DTSTR hybrids included in the regional trials, 33 were high yielders with better adaptability across environments under all stressful and non-stressful testing conditions. Twenty-four of the 33 DTSTR hybrids also yielded well across diverse rainfed environments. The genetic correlations of grain yield under managed drought stress with yield under *Striga-*infestation and multiple rainfed environments were 0.51 and 0.57, respectively. Also, a genetic correlation between yields under *Striga-*infestation with that recorded in multiple rainfed environments was 0.58. These results suggest that the sequential selection scheme offers an opportunity to accumulate desirable stress-related traits in parents contributing to superior agronomic performance in hybrids across stressful and diverse rainfed field environments that are commonly encountered in the tropical savannas of Africa.

## Introduction

Maize is a dominant staple food crop with 70% of its grain used directly for human consumption in sub-Saharan Africa (Vogel et al., 2019). Among the 22 countries in the world where maize provides the highest percentage of calorie in the national diet, 16 are in Africa (Nuss and Tanumihardjo, 2011). However, its low average grain yields that are pervasive in farmers' fields pose a serious threat to food security and livelihoods of millions of farmers in Africa. Farmers face many challenges that affect yields, among which drought and a parasitic weed known as *Striga hermonthica* (Del.) Benth have been recognized as the most widespread stresses limiting the productivity of maize in Africa (Ejeta, 2007; Edmeades, 2013). When drought occurs during flowering of maize, it disrupts pollination and diminishes availability of photosynthate to developing kernels leading to reduction in kernel number and final yield loss of 17 to 60% (Edmeades et al., 1999; Cattivelli et al., 2008; Aslam et al., 2015). Likewise, maize plants with attached *Striga* plants to their roots exhibit stunted growth resulting from the withdrawal of water, nutrients and assimilate from the host by the parasite (Gurney et al., 1999) causing yield loss of up to 100% in severely infested fields in Africa (Kim et al., 2002; Ejeta, 2007). Moreover, the projected rising temperatures and uncertainties in rainfall patterns associated with climate change will further accentuate the intensity and frequency of drought in many parts of Africa (Masih et al., 2014; Shiferaw et al., 2014) and create ideal conditions for *S. hermonthica* to thrive and expand its distribution into suitable new habitats (Mohamed et al., 2007).

Cereal crops grown in the tropical savannas of sub-Saharan Africa (SSA) are often exposed to a combination of drought stress and *S. hermonthica* infestation. When drought occurs in maize fields infested with *S. hermonthica,* it accentuates parasite damage leading to greater yield loss (Adetimirin et al., 2000). Similarly, maize plants infected with *S. hermonthica* produce more abscisic acid (ABA) that trigger stomatal closure to minimize water loss and this can exacerbate the negative effect of drought on productivity of maize (Taylor et al., 1996; Taylor et al., 1998). As both drought stress and *Striga* infection increase ABA synthesis, Ronald et al. (2017) postulates that the ABA-driven increase in stomatal closure under the combined stresses diminishes the uptake of carbon dioxide and the production of photosynthate to sustain plant growth and development. Therefore, approaches that minimize the deleterious effect of the concurrent onslaught of drought and *S. hermonthica* in maize are needed to improve food security and income of farmers who depend on the crop for their livelihoods.

Over many years, breeders in the International Institute of Tropical Agriculture (IITA) have run independent breeding programs with specific emphasis on developing either drought tolerant or *Striga* resistant maize germplasm using reliable screening protocols established for each stress. Significant achievements have been recorded in generating maize varieties that are adapted either to drought stress (Edmeades, 2013; Aslam et al., 2015) or *S. hermonthica* infection (Kim, 1996: Kling et al., 2000; Menkir et al., 2007). Nonetheless, such an approach will be inadequate for areas affected by concurrent presence of drought stress and *S. hermonthica* infestation (Mittler and Blumwald, 2010; Atkinson and Urwin, 2012). Numerous recent reviews report that abiotic and biotic stress combinations inflict greater damage to plant growth, development, and productivity in comparison to each stress applied separately in different crops (Mittler, 2006; Atkinson and Urwin, 2012; Atkinson et al., 2013; Narsai et al., 2013; Prasch and Sonnewald, 2013; Kissoudis et al., 2014; Rejeb et al., 2014; Sewelam et al., 2014; Suzuki et al., 2014; Pandey et al., 2015; Pandey et al., 2017). Consequently, a breeding approach that enhances tolerance of maize to the co-occurrence of drought and *S. hermonthica* can reduce risks and bolster on-farm productivity in tropical savannas.

Mittler (2006) considers simultaneous application of abiotic and biotic stresses critical to accurately characterize the response of crop plants to multiple stresses. However, the difficulty in defining the appropriate timing, duration, and intensity of simultaneous application of drought stress and *S. hermonthica* infestation that mimics the actual conditions occurring in the field has hampered the establishment of a precise screening protocol to select maize germplasm with tolerance to stress combinations (Suzuki et al., 2014; Ramegowda and Senthil-Kumar, 2015). Breeder in IITA have therefore adopted a sequential selection scheme that initially screens early generation lines under artificial *S. hermonthica* infestation during the main cropping season which is then followed by screening the selected lines under managed drought stress during the dry season to develop maize inbred lines with tolerance to stress combinations. Repeated selection of the lines for several pedigree generations using the sequential selection scheme led to the development of homozygous lines with tolerance to drought and resistance to *S. hermonthica* (DTSTR).

The DTSTR maize inbred lines have been evaluated in hybrid combinations through successive stages under artificial *Striga* infested and non-infested conditions as well as under managed drought stress and non-stress conditions until high yielding and multiple stress tolerant elite hybrids (DTSTR) are selected for dissemination to partners for regional testing. Studies are therefore needed to assess the extent to which the sequential selection scheme can stack tolerance to the two stresses in individual maize hybrids. The need for approaches that facilitate the development of crop cultivars with tolerance to abiotic and biotic stresses combinations have become active areas of recent research reviews (Atkinson et al., 2013; Kissoudis et al., 2014; Rejeb et al., 2014; Pandey et al., 2015; Sinha et al., 2016; Pandey et al., 2017). In this context, testing the agronomic performance of elite DTSTR hybrids encapsulating the impacts of the sequential selection scheme relative to commercial hybrids is important to determine the potential that exists in identifying hybrids with tolerance to stress combinations. The present study was therefore conducted to assess the responses of these hybrids to independently applied managed drought stress and artificial *Striga* infestation and examine to what extent maize hybrids that simultaneously express tolerance to these stresses are developed. We also evaluated the responses of the DTSTR and commercial hybrids to multiple rainfed field environments and investigated the relationships of hybrid performance under the two controlled stress conditions with performance in multiple field environments.

## Materials and Methods

### Genetic Materials

Five independent regional trials consisting of 40, 42, 48, 48, and 44 hybrids were evaluated under *Striga* infested and non-infested conditions, managed drought stress (MDS) and full irrigation (WW) as well as in several testing locations under rainfed conditions in 2012, 2013, 2014, 2015, and 2016, respectively. The year-to-year variation in the number of hybrids included in these trials was caused by constant addition of new hybrids and removal of inferior ones. Altogether, 77 three-way cross DTSTR (H01-H77), two *Striga* resistant (H78-H79), and 19 conventional commercial hybrids (H80-H98) plus a local maize variety (H99) were included in the regional trials (**Supplementary Table S1**). The 77 three-way cross DTSTR hybrids were formed from inbred lines that had undergone through eight generations (S8) of inbreeding with sequential selection first under artificial *Striga* infestation at Abuja and Mokwa followed by selection under managed drought stress conditions at Ikenne. The two *Striga* resistant and 19 conventional commercial hybrids obtained from Premier Seeds Nigeria Ltd and SeedCo represent top-crosses, single-crosses, and three 3-way crosses that are marketed in Nigeria and other countries in Africa. The local maize variety (Local) was a farmer preferred recycled hybrid or an improved open-pollinated maize variety commonly grown around the testing site where the regional trials were conducted. The two *Striga* resistant commercial hybrids, which are hereafter referred to as STR hybrids, were developed at IITA but were not specifically bred for tolerance to drought stress. The 19 conventional commercial hybrids as well as the local maize variety, which are hereafter referred to as non-DTSTR hybrids, were not specifically bred for tolerance to drought and resistance to *Striga*. The STR and non-DTSTR hybrids were included in the regional trials as benchmarks against which the performances of the DTSTR hybrids were compared under stressful and non-stressful growing conditions. Among the 99 hybrids included in the regional trials, 17 were tested for 5 years, 17 were tested for 4 years, 20 were tested for 2 years, and the remaining 45 were tested for 1 year only.

The 77 DTSTR hybrids (H01 to H77) were formed from drought tolerant and *Striga* resistant maize inbred lines (**Supplementary Table S1**) developed using a sequential selection scheme in which the inbred lines derived from bi-parental crosses between elite drought tolerant and *Striga* resistant lines as well as narrow and broad-based populations were first screened under artificial *Striga* infestation followed by evaluation of the selected lines under managed drought stress until homozygous lines were developed. Promising lines were continually selected for synchronous time in pollen shed and silking, low ear placement, and other desirable agronomic features first under artificial *Striga* infestation and followed by selection under managed drought stress conditions through eight generation (S8) of self-pollination. The genetic backgrounds of the broad-based source populations (TZLComp1 and STRLowEmergPool), synthetics (ACRSYN-W and IWD-SYN-STR), and a backcross containing *Zea diploperennis* (ZDiploBC4) and their improvements under artificial *Striga* infestation had been extensively described by Kling et al. (2000). The synthetics and populations were also improved for tolerance to drought under managed drought stress using an S_1_ recurrent selection scheme.

### Performance Testing Under Managed Drought Stress and Well-Watered Conditions

The 40, 42, 48, 48, and 44 hybrids included in the five independent regional trials were arranged in 5x8, 7x6, 6x8, 6x8, and 11x4 alpha lattice designs, respectively, and were evaluated with three replications under managed drought stress (MDS) and full irrigation (WW) at the IITA experiment station in Ikenne (6^0^53' N, 3^0^42' E, altitude of 60 m) during the 2011/2012, 2012/2013, 2013/2014, 2014/2015, and 2015/2016 dry seasons. The trials were planted on 21 November in 2011 and on 18 November in 2012, 2013, 2014, and 2015 in two adjacent blocks separated by four ranges each being 4.25 m wide. The four ranges were planted to a commercial hybrid to minimize lateral movement of water from irrigated plots to drought stress plots. The soil at Ikenne is eutric nitosol (FAO classification) and the station has uniform experimental fields. One of the blocks received full irrigation (WW) every week through a sprinkler irrigation system from planting until the hybrids attained physiological maturity. In the second block, drought stress (MDS) was induced by withdrawing irrigation from 35 days after planting to harvesting time of the trials.

Each hybrid was planted in two 4 m long row plots with 0.75 m spacing between rows and 0.25 m spacing between plants within a row. We planted two seeds in each hill and thinned them later to one plant after emergence to attain a population density of 53,333 plants per ha. At the time of sowing, we applied 60 kg N, 60 kg P, and 60 kg K ha^−1^ fertilizer with an additional 60 kg N ha^−1^ fertilizer applied 4 weeks later. The trial field was sprayed with gramazone and atrazine as pre-emergence herbicides at the rate of 5 liters ha^−1^, which was followed by manual weeding to keep the trials weed-free.

### Performance Testing Under Artificial *Striga* Infestation and Non-Infested Conditions

The regional trials were also evaluated under artificial *S. hermonthica* infestation (STRIN) and non-infested conditions (STRNO) at Kubwa and Mokwa in Nigeria during the main rainy seasons for 5 years using the alpha lattice designs described above with two replications. Kubwa and Mokwa are located 340 km apart and represent different climatic conditions. Kubwa is located near Abuja (9°14'N and 7°35'E, 445 m elevation), has a ferric luvisol Plinthustalf soil containing 81% sand, 12% silt, and 7% clay, receives about 1,389 mm of rainfall, and had average monthly minimum temperatures of 19 to 24^0^ C as well as average monthly maximum temperatures of 26 to 34^0^ C during evaluation of the hybrids for 5 years (**Supplementary Table S2**). At Kubwa, the growing season starts in May and ends in October. Mokwa is situated in Niger state (9°29'N and 5°05'E, 153 m elevation), has a Tropeptic Haplustox soil that is fine and kaolinitic in nature, receives 1,150 mm of rainfall and had average monthly minimum temperatures of 20 to 23^0^ C as well as average monthly maximum temperatures of 29 to 34^0^ C during evaluation of the hybrids for 5 years (**Supplementary Table S2**). The growing season at Mokwa starts at the end of June and ends in October.

Each hybrid was planted in adjacent infested and non-infested strips facing opposite to each other and separated by 1.5 m alley. Within each strip, the same hybrid was planted in two infested rows and two non-infested rows that were planted directly opposite to each other to determine precise estimates of yield losses due to *S. hermonthica* damage (Kling et al., 2000). Each row was 5 m long with spacing of 0.75 m between rows and 0.25 m spacing between plants within a row. The non-infested rows were treated with ethylene, which was a gas injected into the soil from a cylinder to stimulate the germination of *Striga* seeds 2 weeks before planting, to eliminate any potential *S. hermonthica* seeds present in the soil. Every year, *S. hermonthica* seeds were collected from farmers' sorghum fields around Abuja and Mokwa and used for infestation, which was carried out by injecting 8.5 g of sand-mixed *S. hermonthica* seed inoculum into holes of about 6 cm deep and 10 cm wide. The number of germinable *S. hermonthica* seeds placed in each hill was estimated at 3,000. We placed two maize seeds into each hole infested with sand-mixed *S. hermonthica* seeds and covered them with soil. One plant was manually removed from each hill 2 weeks after planting (wks) to attain a population density of 53,333 plants ha^−1^. As *S. hermonthica* infection is high under low nitrogen (Kling et al., 2000), nitrogen, phosphorus and potassium were applied at the rate of 30 kg ha^−1^, 60 kg ha^−1^, and 60 kg ha^−1^ at planting, respectively, and additional 30 kg ha^−1^ nitrogen was applied 4 weeks later. Weeds other than *S. hermonthica* were removed by hand throughout the cropping season.

### Performance Testing in Multi-Environment Trials Under Rainfed Conditions (MET)

**Figure 1:**
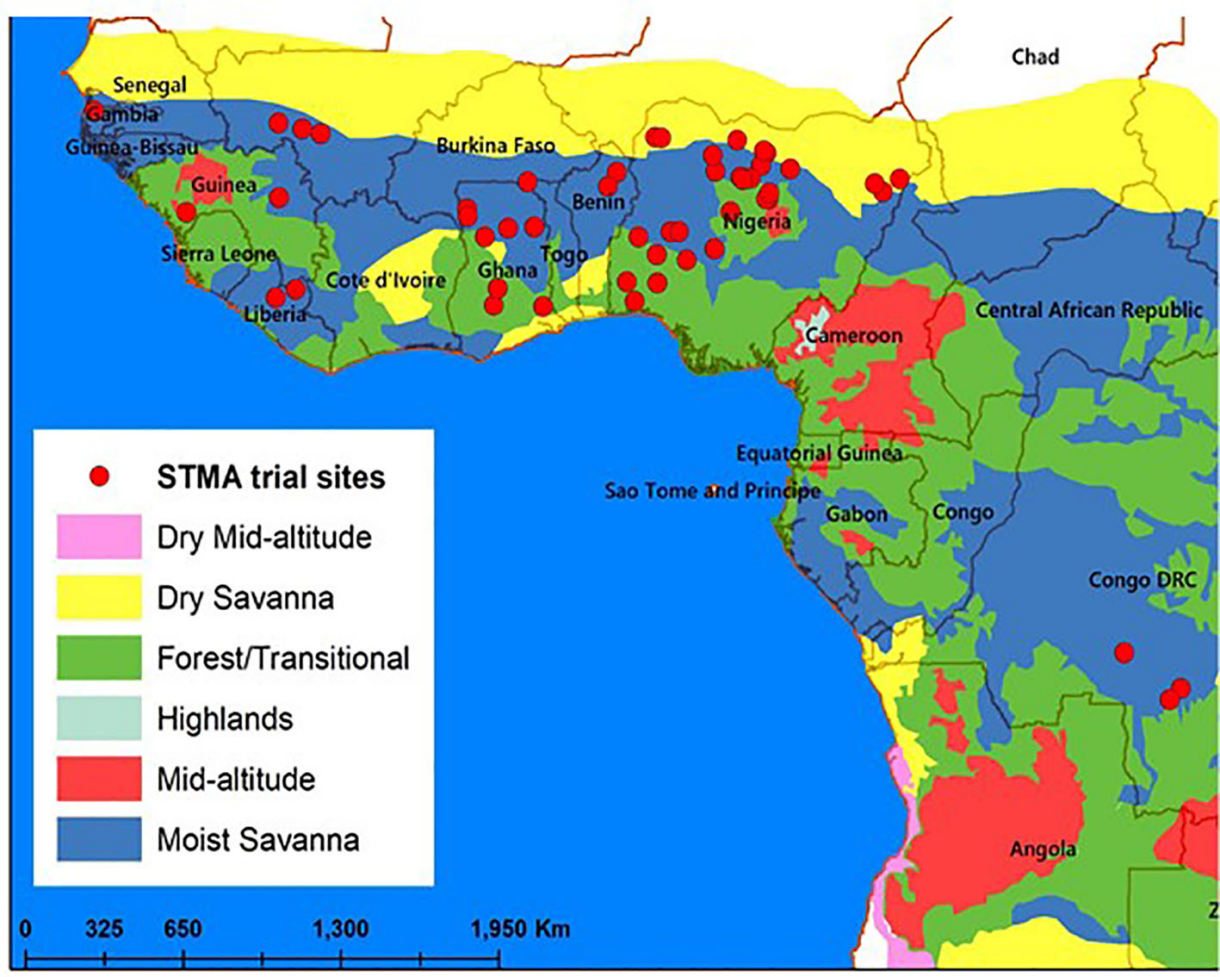
Testing sites used for running the regional trials in 2012, 2013, 2014, 2015, and 2016.

The regional elite DTSTR hybrid trials were also arranged in alpha lattice designs described above with three replications and evaluated during the main rainy seasons in collaboration with the national agricultural research systems (NARS) and private seed companies in 35 locations in 2012, 25 locations in 2013, 26 locations in 2014, 23 locations in 2015, and 16 locations in 2016 in nine countries in West Africa (**Figure 1**). These test locations represent the diverse maize growing environments in this region. Each hybrid was planted in two rows each 5 m long with spacing of 0.75 m between rows and 0.5 m between plants within a row. The collaborators in the NARS and private seed companies used crop management practices, rates of fertilizer application, and weed control methods recommended for each of their testing location when they conducted these trials.

### Trait Measurements

Even though several traits were measured in the regional trials evaluated under MDS, WW, STRIN, STRNO, and in MET, grain yield was considered as the primary trait for analyses in the present study because direct selection for this trait under stressful conditions allows identification of promising stress tolerant hybrids (Verulkar et al., 2010). All ears harvested from each plot were shelled to determine percent moisture, which was used to determine grain yield adjusted to 15% moisture under each growing condition. Grain yield was calculated from shelled grain under MDS and WW, and from ear weight and grain moisture under STRIN, STRNO, and in MET assuming a shelling percentage of 80% and final adjusted moisture content of 15% in each testing environment.

### Statistical Analysis

The year-to-year variation in the number of hybrids included in the five regional trials created unbalanced data sets for grain yield recorded in each location. Year-location combinations are hereafter referred to as environments. These data sets for MDS, WW, STRIN, STRNO, and MET were subjected to analyses of variance and covariance using a mixed model with the restricted maximum likelihood procedure (REML) of SAS (Vargas et al., 2013). In these analyses, all effects including hybrids were considered random and best linear unbiased predictors (BLUPs) were computed. The hybrids were considered random because they represent the elite DTSTR hybrids developed and disseminated to partners for regional testing in our breeding program. The significance test of the effects of the variance parameters was obtained from the mixed model analysis using PROC Mixed in SAS (SAS Institute, 2016). Variance component estimates from the mixed model were used to calculate repeatability (broad sense heritability) values, the grand means, and the coefficient of variation (CV) (Vargas et al., 2013). Phenotypic and genotypic correlations between yield BLUPs were computed for pairs of growing conditions (MDS, WW, STRIN, STRNO, and MET) to determine the similarity or differences in response patterns of hybrids using the procedure described by Alvarado et al. (2015) in META-R.

Descriptive statistics were computed for yield BLUPs of the DTSTR, STR, and non-DTSTR hybrids recorded under MDS, WW, STRIN, and STRNO as well as in MET using the univariate procedure of SAS (SAS Institute, 2016). Hybrid yield BLUPs of individual environments (years or year-location combinations) calculated for each stressful or non-stressful testing condition were subjected to canonical discriminant analyses to summarize the differentiation between the three hybrid groups using the CANDISC procedure in SAS (SAS Institute, 2016). The pair-wise squared distance between hybrid groups (DTSTR, STR, and non-DTSTR) was tested for significance using Mahalanobis distance statistics (Mahalanobis, 1936).

The unbalanced yield data obtained from the regional trials conducted under MDS, WW, STRIN, STRNO, and in MET for 5 years were then subjected to mixed model analyses using a factor analytic model (FA) with covariance structure. While environments were considered as fixed effects in these analyses, replicates within environments, hybrids and hybrid by environment interaction were regarded as random effects (Piepho, 1998; Smith et al., 2001). This approach has proven to be effective in generating biplots which graphically represent genotype stability and adaptability in MET (Burgueño et al., 2008; Crossa et al., 2010; Crossa et al., 2013; Figueiredo et al., 2015). The two factor analysis (FA(2)) that has become a standard model for MET analysis (Kelly et al., 2007) was used in the analyses of all data sets. The resulting two factors (FA1 and FA2) scores were utilized for assessing the hybrid by environment relationships and the adaptability and stability of hybrids in our study. Hybrids with large positive FA1 scores could be regarded as high yielders with desirable ranks across years (Stefanova and Buirchell, 2010; Smith et al., 2015), whereas those with positive FA1 scores but with close to zero FA2 scores under each growing condition were regarded as productive and stable hybrids (Nuvunga et al., 2015). To discern the adaptability patterns of the DTSTR hybrids relative to the commercial hybrids, yield BLUP estimates from each hybrid was regressed against the first factor (FA1) scores using PROC REG in SAS (Smith et al., 2001).

Among the 99 hybrids, 14 DTSTR and two STR commercial hybrids plus the farmer preferred local maize variety were continuously evaluated for 5 years. These hybrids were used to assess consistency of hybrid yield BLUP estimates across years/environments under controlled stressful (MDS and STRIN) and non-stressful (WW and STRNO) growing conditions as well as in MET. Yield BLUPs of these genotypes were ranked in each year/environment using PROC RANK in SAS (SAS Institute, 2016). The resulting ranks were then analyzed for concordance across years/environments under all testing conditions (Kendall, 1962). To further determine consistency in average performance of the hybrid groups under each testing condition (MDS, WW, STRIN, STRNO, and MET), analysis of the yield BLUPs of the 17 hybrids for each year or environment was carried out using the Statistical Analysis System (SAS) software version 9.4 (SAS Institute, 2016). Analysis of variance (ANOVA) was performed using the general linear procedure (Proc GLM). Following this analysis, the;east squares means/PDIFF (LSMEANS/PDIFF) option at *P* |t|< 0.05 was used to test the significant differences among the average grain yields of the different hybrid groups (DTSTR, STR, and Local).

## Results

### Hybrid Performance Under Managed Drought Stress and Fully Irrigated Conditions

The rainfall recorded during the period in which the regional trials were evaluated at Ikenne was very low, except for 2012/2013 that received an appreciable rainfall in November and February (**Supplementary Figure S1**). As a result, the hybrids were exposed to water deficit for 35 to 40 days prior to flowering with no additional irrigation applied thereafter. Relative to fully irrigated condition, the drought stress attained in this study reduced average hybrid yield by 66% in 2012, 18% in 2013, 62% in 2014, 72% in 2015, and 79% in 2016. As shown in **Table 1**, significant differences in grain yield were observed among hybrids and their interaction with years but not among testing years under both MDS and WW conditions. Repeatability estimates for grain yield were 0.70 for MDS and 0.82 for WW conditions (**Supplementary Table S3**). The range of mean grain yields of the DTSTR hybrids was broader than those of the STR and non-DTSTR commercial hybrids under these testing conditions (**Table 2**). As a group, the DTSTR hybrids produced significantly higher average grain yields than the STR and non-DTSTR hybrids under MDS and WW conditions (**Supplementary Table S4**). In contrast, the non-DTSTR hybrids had significantly higher average grain yield than the STR hybrids under WW but not under MDS.

**Table 1:** Covariance estimates from a mixed model analysis with the restricted maximum likelihood procedure for 5 years.

Parameters	Covariance estimates	Standard error	Z Value	Pr > Z
	Grain yield under drought stress			
Year	672319	482707	1.39	0.0818
Rep (year)	9358.63	8038.53	1.16	0.1222
Hybrid	90963	30850	2.95	0.0016
Year*hybrid	128557	28932	4.44	<.0001
Residual	206380	18072	11.42	<.0001
	Grain yield under full irrigation			
Year	414959	301863	1.37	0.0846
Rep (year)	936.6	5577.62	0.17	0.4333
Hybrid	424238	103602	4.09	<.0001
Year*hybrid	268496	61127	4.39	<.0001
Residual	401373	34979	11.47	<.0001

**Table 2:** Descriptive statistics for yield BLUPs estimated from regional trials evaluated under managed drought stress and full irrigation at Ikenne during the dry season for 5 years.

Hybrid groups	Number of hybrids	Minimum	Maximum	Mean
	Drought stress			
DTSTR hybrids	77	1076	2068	1707 ± 19
STR commercial hybrids	2	1378	1503	1441 ± 63
Non-DTSTR commercial hybrids	20	1030	1918	1501 ± 50
	Fully irrigated			
DTSTR hybrids	77	2878	5233	4348 ± 51
STR commercial hybrids	2	3648	3668	3658 ± 10
Non-DTSTR commercial hybrids	20	2668	4984	3833 ± 119

**Figure 2:**
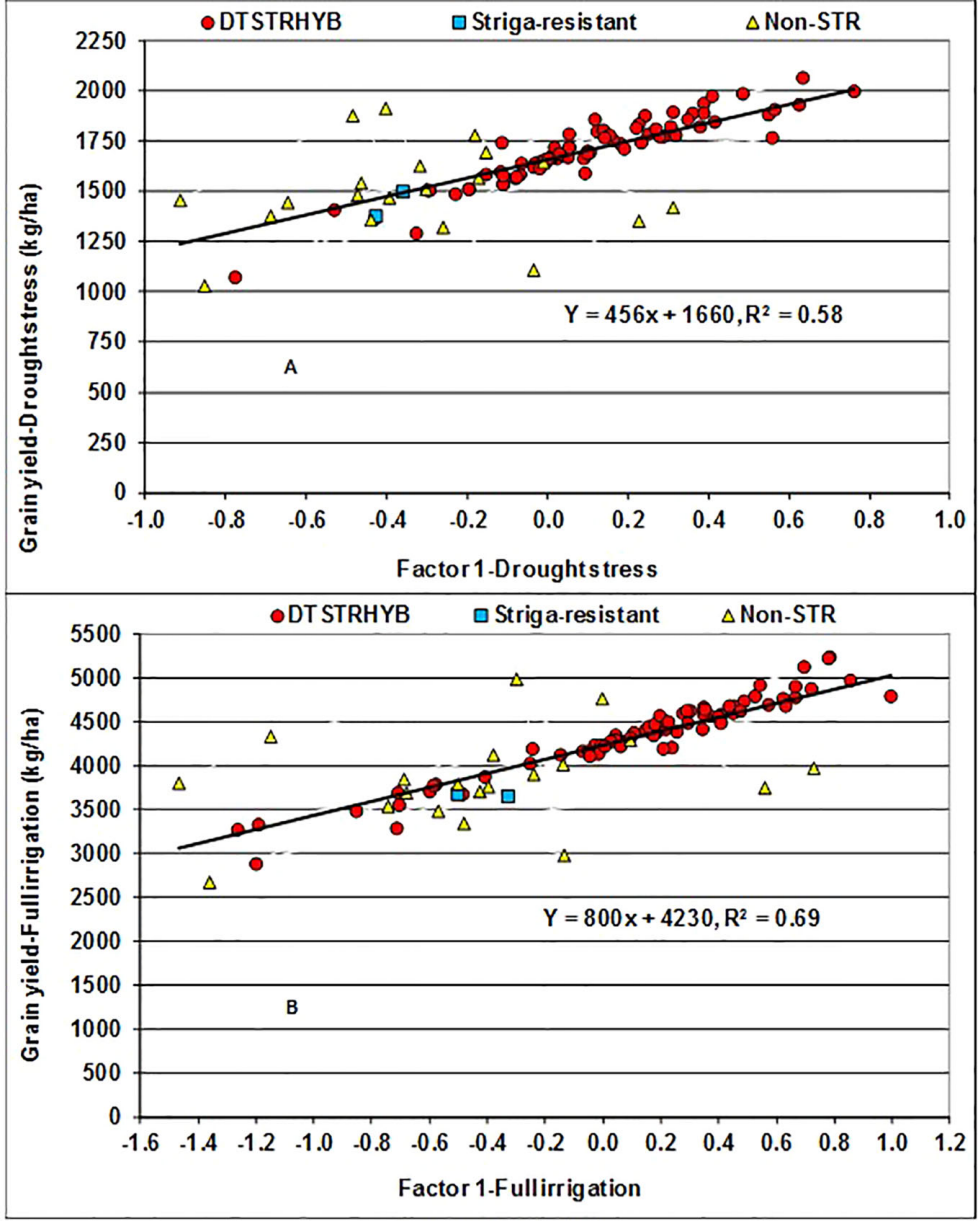
Regression of Best Linear Unbiased Predictors (BLUPs) of grain yield on the first factor (FA1) scores of hybrids evaluated under drought stress **(A)** and fully irrigated conditions **(B)** for five years.

In the FA(2) model, the first factor (FA1) alone captured a total variation of 52% under MDS and 73% under WW condition, allowing appropriate assessment of adaptability of hybrids. As all the estimated FA1 loadings were positive varying from 0.31 to 1.31 under MDS and from 0.47 to 1.84 under WW, the hybrids with large positive FA1 scores could be regarded as high yielders with desirable ranks across years (Stefanova and Buirchell, 2010; Smith et al., 2015). The results of regression analyses showed that for every unit increase in FA1 score, yield BLUP estimates increased by 456 kg ha^−1^ under MDS and 800 kg ha^−1^ under WW conditions (**Figure 2**). Under MDS, only 26 DTSTR hybrids had negative FA1 scores and relatively lower grain yields (**Supplementary Tables S3** and **S4**). In contrast, 20 of the 22 commercial hybrids had negative FA1 scores and relatively lower grain yields. It is interesting to note that six non-DTSTR hybrids with negative FA1 scores produced grain yields varying from 1629 to 1918 kg ha^−1^ under MDS, possibly due to the selection of these hybrids based on wide area testing. Under WW condition, only 21 DTSTR hybrids had negative FA1 scores and produced grain yields varying from 2878 to 4237 kg ha^−1^ whereas 18 commercial hybrids showed negative FA1 scores and produced grain yields varying from 2668 to 4984 kg ha^−1^. Among the 77 DTSTR hybrids, 44 had close to zero or positive FA1 scores under both MDS and WW condition (**Supplementary Table S5**). We found a few DTSTR hybrids having positive FA1 scores that also had close to zero FA2 scores under both MDS and WW conditions that could be regarded productive and stable (**Supplementary Figure S2**). In contrast, there was only one commercial hybrid that had a positive FA1 score combined with close to zero FA2 score under MDS but not under WW conditions.

### Hybrid Performance Under Artificial *Striga* Infestation and Non-Infested Conditions

The environmental average grain yields for regional trials varied from 1727 kg ha^−1^ to 4244 kg ha^−1^ under STRIN and from 2472 kg ha^−1^ to 5525 kg ha^−1^ under STRNO conditions. A yield loss of 65% was recorded under STRIN in a commercial hybrid that was not bred for resistance to S*. hermonthica*, indicating that the level of *Striga* infection was severe in the present study. Overall yield reductions from *Striga* damage varied from 3% to 50% for DTSTR hybrids, from 10% to 15% for STR hybrids, and from 29% to 65% for non-DTSTR hybrids (**Supplementary Table S3**). About 64% of the DTSTR hybrids sustained yield losses of less than 20%. In the analysis of covariance, grain yield was significantly affected by the environment, rep (environment), hybrid, hybrid x environment interaction, and residuals under both STRIN and STRNO conditions (**Table 3**). Repeatability estimates for grain yield were strikingly high for both STRIN (0.93) and STRNO (0.88) conditions (**Supplementary Table S3**). Grain yields of the DTSTR hybrids had a wider range as opposed to the STR and non-STR hybrids under both STRIN and STRNO conditions (**Table 4**). On average, the DTSTR hybrids had significantly higher grain yields in comparison to the non-DTSTR hybrids but not the STR hybrids under both STRIN and STRNO conditions (**Supplementary Table S6**). Also, the STR hybrids had significantly greater average grain yields than the non-DTSTR hybrids under the two testing conditions.

**Table 3:** Covariance estimates from a mixed model analysis with the restricted maximum likelihood procedure for grain yield of hybrids recorded at two locations under *Striga* infestation and non-infested conditions for 5 years.

Parameters	Covariance estimates	Standard error	Z Value	Pr > Z
	Grain yield under *Striga* infestation			
Environment	527972	266695	1.98	0.0239
Rep (environment)	55266	29482	1.87	0.0304
Hybrid	703126	122601	5.74	<.0001
Environment*hybrid	186906	45514	4.11	<.0001
Residual	729203	45601	15.99	<.0001
	Grain yield under non-infested condition			
Environment	1071044	525386	2.04	0.0207
Rep (environment)	70997	35690	1.99	0.0233
Hybrid	362084	79665	4.55	<.0001
Environment*hybrid	183167	45828	4	<.0001
Residual	714689	44953	15.9	<.0001

**Table 4:** Descriptive statistics for yield BLUPs estimated from regional trials evaluated under *Striga* infestation and non-infested conditions at Abuja and Mokwa in Nigeria during the main cropping season for 5 years.

Hybrid group	Number of hybrids	Minimum	Maximum	Mean
	*Striga* infestation			
DTSTR hybrids	77	1973	4390	3391 ± 65
STR commercial hybrids	2	3379	3686	3533 ± 154
Non-DTSTR commercial hybrids	20	1161	3186	2079 ± 101
	Non-infested conditions			
DTSTR hybrids	77	2716	4932	4137 ± 53
STR commercial hybrids	2	3962	4117	4040 ± 78
Non-DTSTR commercial hybrids	20	2877	4633	3698 ± 118

**Figure 3:**
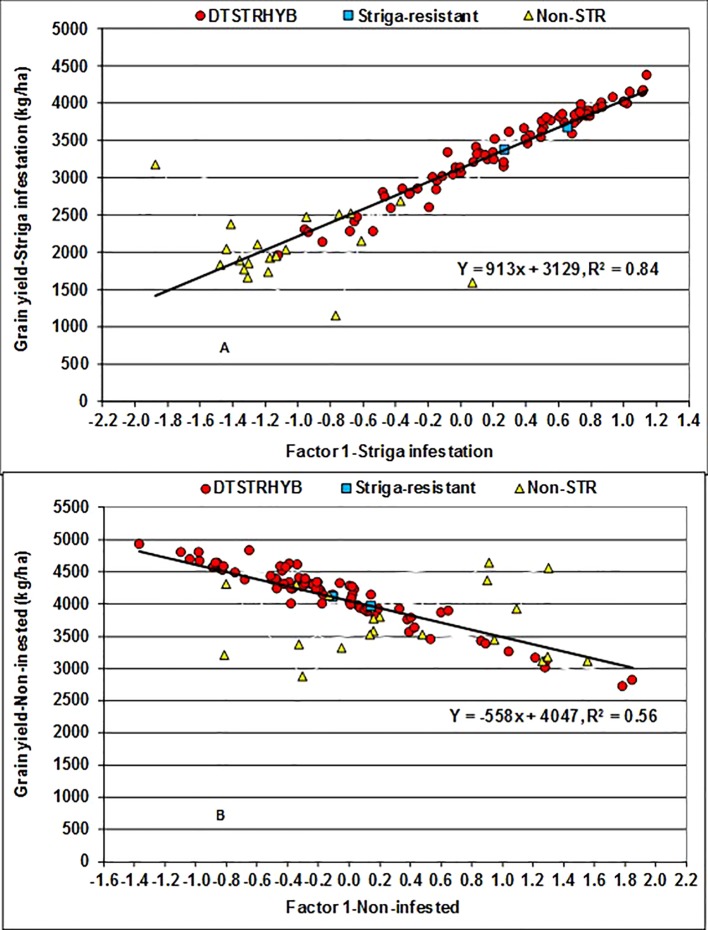
Regression of Best Linear Unbiased Predictors (BLUPs) of grain yield on the first factor (FA1) scores of hybrids evaluated under artificial *Striga* infested **(A)** and non-infested **(B)** conditions at two locations for five years.

The FA(2) model accounted for 91% of the total hybrid by environment variation in grain yield recorded under STRIN and 88% under STRNO conditions. FA1 alone represented 82% of the total variation under STRIN and 57% under STRNO conditions. FA1 had positive loadings of 0.60 to 1.39 under STRIN but negative loading of −0.97 to −017 under STRNO conditions. As a result, the hybrids with large positive FA1 scores under STRIN and negative FA1 scores under STRNO condition could be considered as high yielders with favorable ranks across environments (Stefanova and Buirchell, 2010). The regression analyses of yield BLUP estimates on FA1 scores accounted for 84% of the total variation among hybrids under STRIN and 56% under STRNO (**Figure 3**). In these analyses, every unit increase in FA1 score was associated with 913 kg ha^−1^ increase in grain yield under STRIN but with 558 kg ha^−1^ decrease in grain yield under STRNO condition. Among the 99 hybrids, 53 DTSTR and two STR commercial hybrids produced more than 3000 kg ha^−1^ yield and had close to zero or positive FA1 scores, whereas 19 non-DTSTR hybrids had negative FA1 scores and produced less than 3,000 kg ha^−1^ grain yields under STRIN (**Supplementary Tables S3** and **S5**). Again, 46 DTSTR, one STR and three non-DTSTR hybrids had negative FA1 scores and produced more than 4,000 kg ha^−1^ grain yields under STRNO condition. Out of the 77 DTSTR hybrids, 42 had close to zero or positive FA1 scores under STRIN and negative FA1 scores under STRNO conditions (**Supplementary Tables S3** and **S5**). Furthermore, a few DTSTR hybrids that could be considered high yielding and stable combined close to zero FA2 scores with positive FA1 scores under STRIN while at the same time they had close to zero FA2 scores with negative FA1 scores under STRNO condition (**Supplementary Figure S3**). However, none of the non-DTSTR hybrids had a combination of positive FA1 scores and close to zero FA2 scores under both STRIN and STRNO conditions.

### Relationship Between Performances Under Managed Drought Stress and Artificial *Striga* Infestation

We examined the similarity in response patterns of elite hybrids to MDS and STRIN relative to their response to WW and STRNO using phenotypic and genetic correlations. The phenotypic correlations between grain yields measured under MDS and WW (r_p_ = 0.74) and between those recorded under STRIN and STRNO (r_p_ = 0.73) were significant (p < 0.0001) and strong. We also found significant (p < 0.0001) phenotypic correlations between grain yields of MDS and STRIN (r_p_ = 0.42) and those of WW and STRNO (r_p_ = 0.67). In addition, significant (p < 0.01) and strong genetic correlations were found between MDS and WW (r_g_ = 0.91) and between STRIN and STRNO (r_g_ = 0.88). The genetic correlations of yields under MDS with STRIN was moderate (r_g_ = 0.51) but was not significant. As FA1 accounted for the largest proportion of the variation in yield BLUPs in the FA(2) models, the correlation between FA1 scores of MDS and STRIN and between WW and STRNO were used as the bases to further assess the similarity in adaptability of hybrids across different growing environments. The correlations of FA1 scores of MDS with WW (r = 0.74) and those of STRIN with STRNO (r = −0.67) were significant (p < 0.0001). Also, there were significant (p < 0.0001) correlations between FA1 scores of MDS and STRIN (r = 0.51) and those of WW and STRNO (r = −0.74). Out of the 77 DTSTR hybrids evaluated in the present study, 39 had close to zero or positive FA1 scores under both MDS and STRIN (**Supplementary Table S3**). Thirty-three of 39 DTSTR hybrids also had close to zero or positive FA1 scores under WW and close to zero or negative FA1 scores under STRNO conditions.

### Hybrid Performance in Multi-Environment Trials Under Rainfed Conditions

The regional trials evaluated under rainfed environments had mean grain yields varying from 460 kg ha^−1^ to 7,993 kg ha^−1^. Overall, grain yield was affected by the environment, rep (environment), hybrid, hybrid x environment interaction, and the residuals (**Table 5**). The repeatability estimate for yield was very high (0.98) for MET (**Supplementary Table S3**). As a group, the DTSTR hybrids exhibited a wider range in yield BLUP estimates in comparison to the STR and non-DTSTR hybrids (**Table 6**). Also, the DTSTR hybrids produced significantly higher average yield as opposed to the non-DTSTR hybrids but not the STR hybrids in MET (**Supplementary Table S7**).

**Table 5:** Covariance estimates from a mixed model analysis with the restricted maximum likelihood procedure for grain yield of hybrids recorded in diverse rainfed field environments in 2012, 2013, 2014, 2015, and 2016.

Parameters	Covariance estimates	Standard error	Z Value	Pr > Z
Environment	2969448	382897	7.76	<.0001
Rep (environment)	95604	10597	9.02	<.0001
Hybrid	217168	33629	6.46	<.0001
Environment*hybrid	233260	10973	21.26	<.0001
Residual	830634	11703	70.98	<.0001

**Table 6:** Descriptive statistics for yield BLUPs estimated from regional trials evaluated across diverse rainfed field environments (MET) in 2012, 2013, 2014, 2015, and 2016.

Hybrid group	Number of hybrids	Minimum	Maximum	Mean
DTSTR hybrids	77	2360	5097	4326 ± 069
STR commercial hybrids	2	3884	3957	3921 ± 037
Non-DTSTR commercial hybrids	20	2838	5148	4146 ± 147

**Figure 4:**
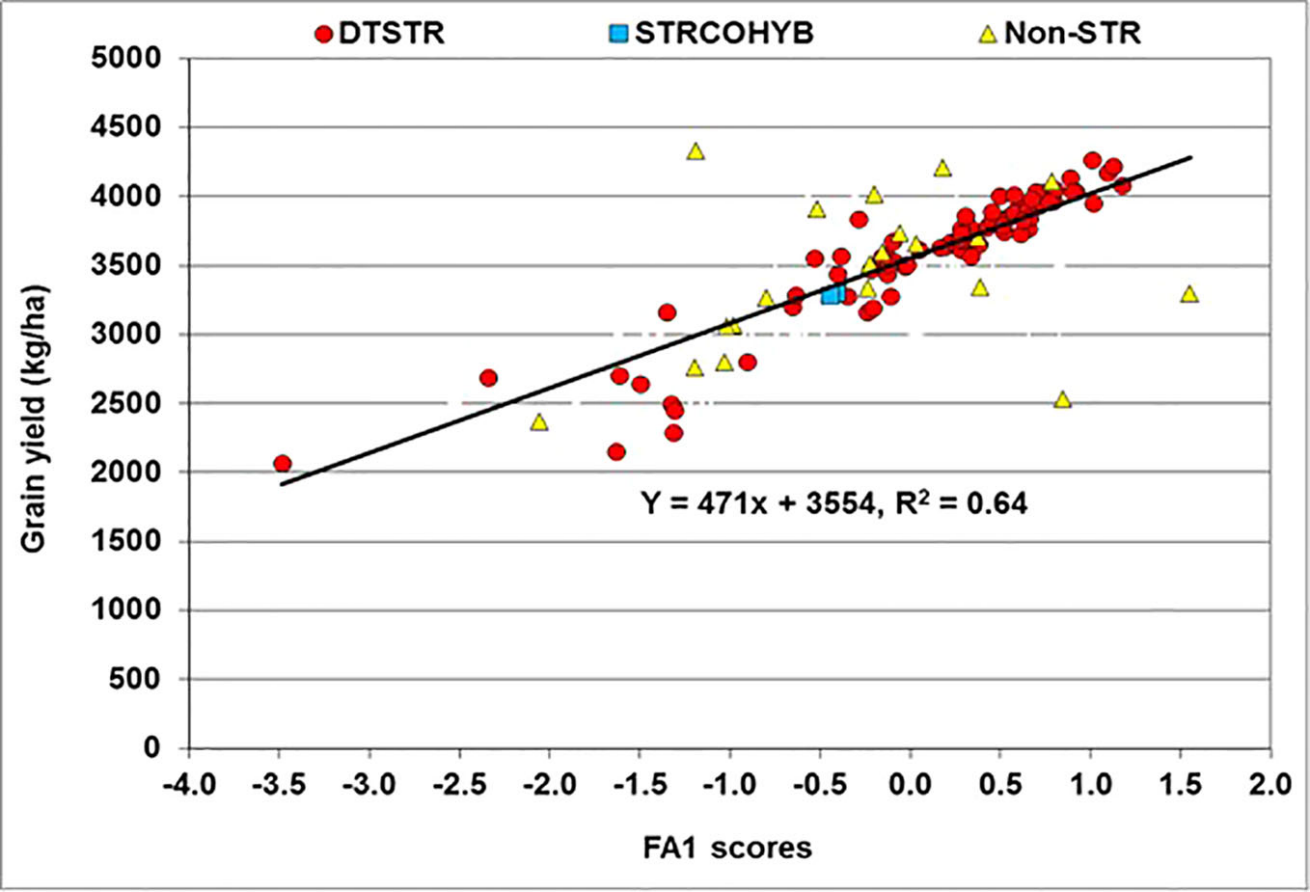
Regression of Best Linear Unbiased Predictors (BLUPs) of grain yield on the first factor (FA1) scores of hybrids obtained from multi-environment trails conducted under rainfed conditions.

The FA(2) model accounted for 77% of the total yield variation in MET. In this model, FA1 represented 53% of the total hybrid x environment variation in grain yield and had positive loadings varying from 0.31 to 1.31. Many hybrids that had large positive FA1 scores in MET were then regarded as high yielding hybrids with better ranks across environments. In the regression analyses of yield BLUP estimates on FA1 scores, the model accounted for 64% of the total variation among hybrids. This analysis showed that for every unit increase in FA1 score grain yield increased by 541 kg ha^−1^ (**Figure 4**). Among the 99 hybrids evaluated in our study, 48 DTSTR and three non-DTSTR hybrids had close to zero or positive FA1 scores and produced above 3,500 kg ha^−1^ yield in MET (**Supplementary Tables S2** and **S4**). We also found some DTSTR hybrids with close to zero FA2 scores and positive FA1 scores (**Supplementary Figure S4**) that could be regarded as high yielding and stable hybrids across diverse rainfed environments. However, none of the non-DTSTR hybrids combined a positive FA1 score with close to zero FA2 score in the MET.

### Relationship of Performances Under Drought Stress and *Striga* Infestation With Performance in Multi-Environment Trials

Yield BLUP estimates for MET had significant (p <0.0001) phenotypic correlation with those of MDS (r_p_ = 0.49), STRIN (r_p_ = 0.51), WW (r_p_ = 0.66), and STRNO (_p_r = 0.81) conditions. The genetic correlations of BLUP estimates for MET with those of WW (r_g_ = 0.76) and STRNO (r_g_ = 0.92) were significant (p < 0.0001) but not with those of MDS (r_g_ = 0.57) and STRIN (r_g_ = 0.58). The correlation between FA1 scores of MET with MDS (r = 0.37), MET with STRIN (r = 0.50), MET with WW (r = 0.58), and MET with STRNO (r =−0.63) were significant (p <0.0001), highlighting the presence of varying levels of similarity in adaptability of hybrids under the presence or absence of specific stress relative to those in multiple field environments. Among the 33 DTSTR hybrids with close to zero or positive FA1 scores under MDS, WW, STRIN, and STRNO, 24 also had close to zero or positive FA1 scores in MET (**Supplementary Table S4**).

### Performance of Selected Hybrids Under Controlled Stresses and in Multi-Environment Trials

**Figure 5:**
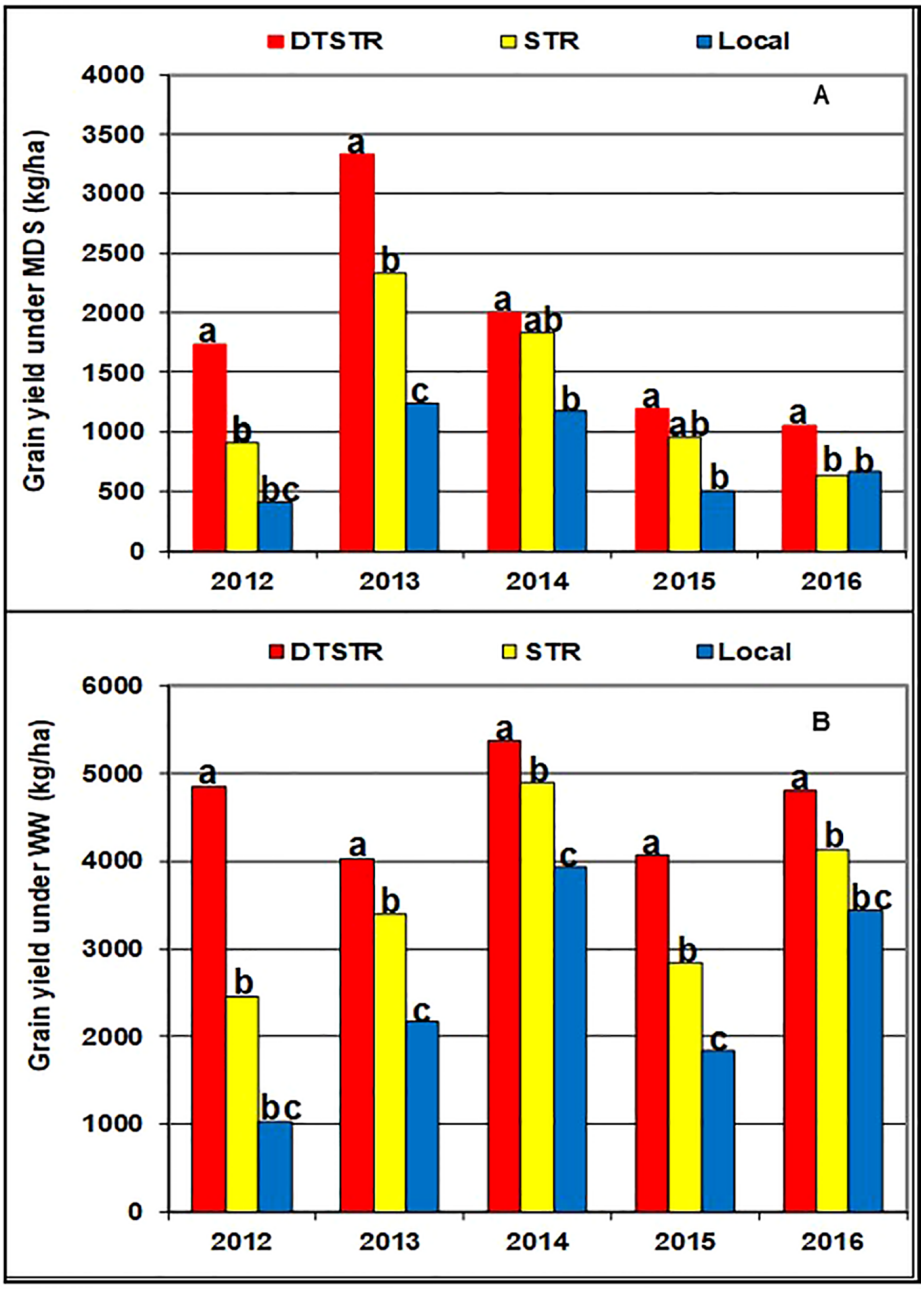
Overall mean grain yields of three sets of hybrids tested under managed drought stress (MDS-**A**) and fully irrigated (WW-**B**) conditions in each of the five years (2012–2016). Average grain yields of the different hybrid groups showing the same lower-case letters were not significantly different from each other at *P* |t|< 0.05 based on the Proc GML and LSMEANS/PDIFF procedures of SAS (SAS Institute, 2016).

**Figure 6:**
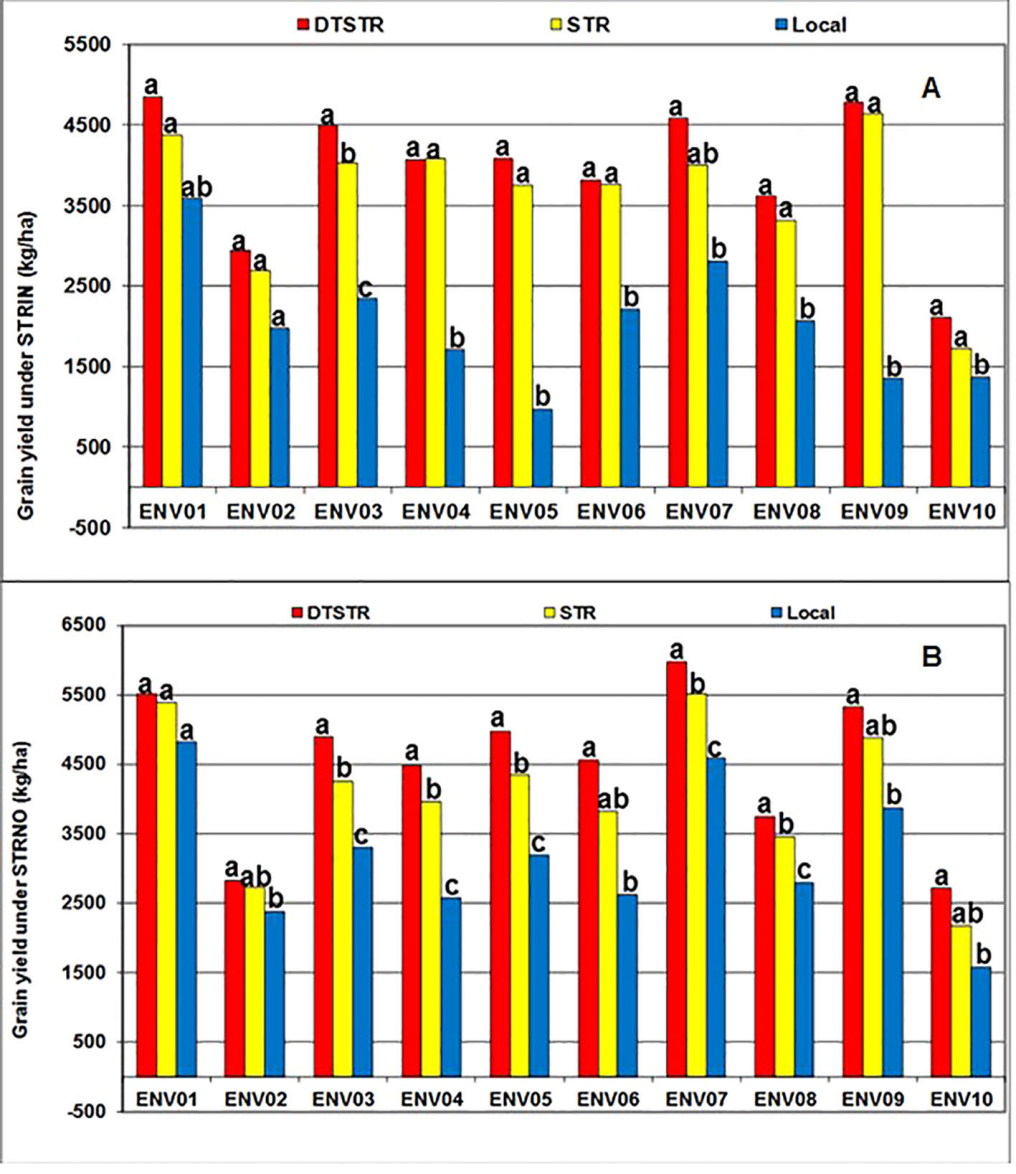
Overall mean grain yields of three sets of hybrids tested under artificial Striga infested (STRIN-**A**) and non-infested (STRNO-**B**) conditions in 10 environments (ENVO1-ENVO10). Average grain yields of the different from each other at *P* |t|<0.05 based on the Proc GLM and LSMEANS/PDIFF procedures of SAS (SAS Institute, 2016).

**Figure 7:**
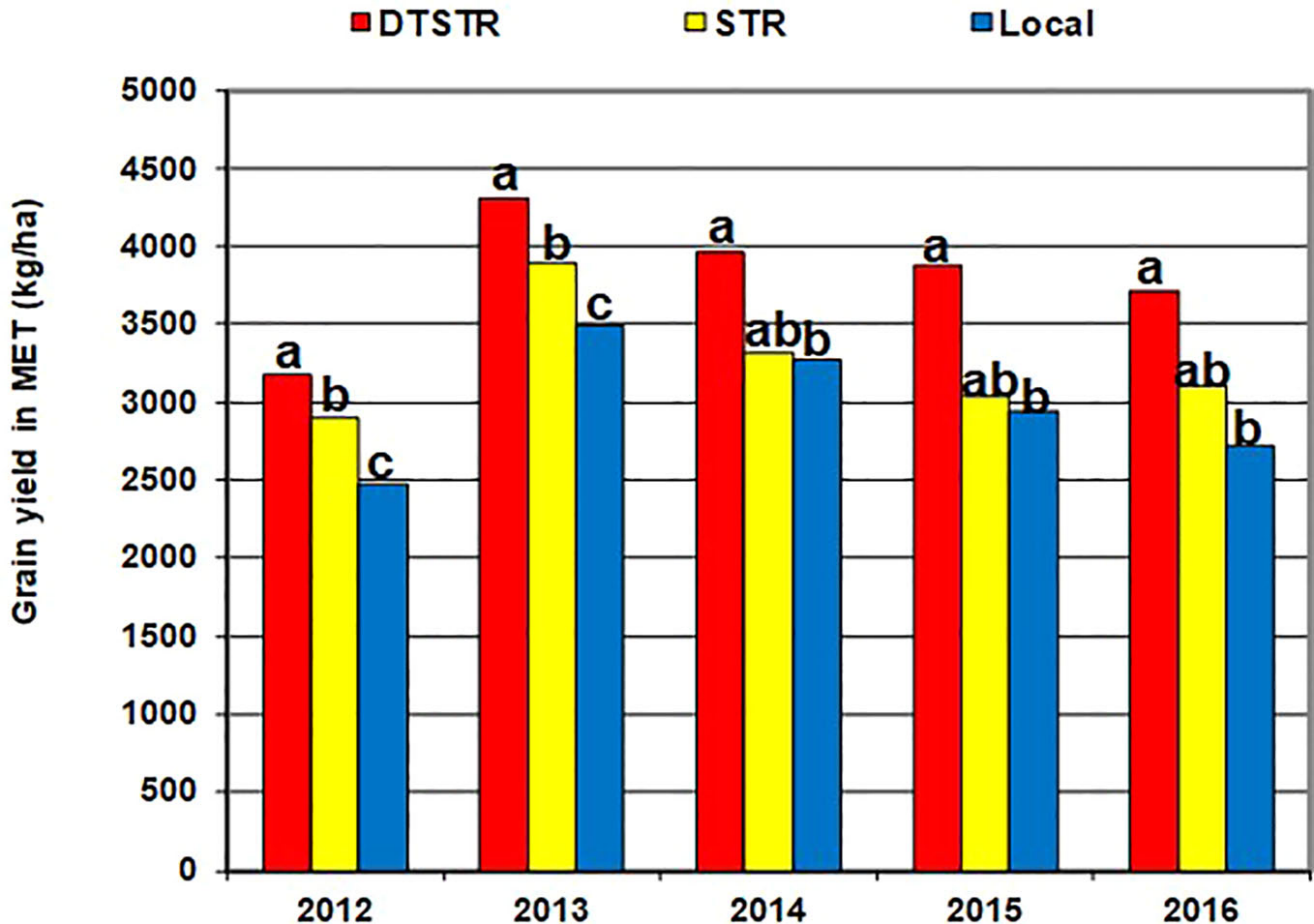
Overall mean grain yields of the different sets of hybrids tested in diverse rainfed field environments (MET) for each of the five years (2012–2016). Average grain yields of the different hybrid groups showing the same lower-case letters were not significantly different from each other at *P* |t| < 0.05 based on the Proc GML and LSMEANS/PDIFF procedures of SAS (SAS Institute, 2016).

Smith et al. (2005) recommend use of 5 to 10 years data to obtain a representative sample of seasons for accurate assessment of varietal performance in diverse growing environments. In our study, 14 DTSTR, two STR commercial hybrids and “a local maize variety”, which were tested for 5 years, were then chosen for assessing yield potential across years/environments under MDS, WW, STRIN, STRNO, and in MET. The average grain yields of the DTSTR hybrids were consistently higher than those of the STR hybrids and the Local maize variety across years under both MDS and WW conditions (**Figure 5** and **Supplementary Tables S8** and **S9**). Also, the DTSTR hybrids on average produced consistently higher grain yields than the Local maize variety but were competitive to the STR hybrids in all environments under both STRIN and STRNO conditions (**Figure 6** and **Supplementary Tables S10** and **S11**). In MET, the average yield of the DTSTR hybrids was always higher than those of the STR hybrids and the Local maize variety across 5 years (**Figure 7** and **Supplementary Tables S12** and **S13**). Further assessment of the coefficients of concordance (W) of the ranks of the 17 hybrids were found to be significant (p < 0.0001) across years under MDS (W = 0.51) and WW (W = 0.84) and across environments under STRIN (W = 0.60) and STRNO (W = 0.62) as well as in MET (W = 0.36). As shown in **Table 7**, the average yield advantages of each of the best seven DTSTR hybrids (H01, H06, H07, H09, H11, H15, and H17) over the best STR commercial hybrid (H79) varied from 21 to 38% under MDS, from 15 to 36% under WW, from 11 to 30% under STRIN, from 7 to 24% under STRNO, and from 12 to 23% in MET (**Table 7**). The best DTSTR hybrids also produced 32 to 115% more grain yields than the Local maize variety under all stressful and non-stressful testing conditions.

**Table 7:** Grain yield BLUPs of selected hybrids constantly evaluated under managed drought stress and fully irrigated conditions, artificial *Striga* infestation and non-infested conditions as well as in diverse rainfed field environments for 5 years.

Hybrid	Grain yield (kg ha^−1^)				
	Managed drought stress	Fully irrigated	*Striga* infested	*Striga* non-infested	Multiple rainfed field environments
H01	2068	4578	3746	4374	3857
H06	1988	4682	4390	4932	4031
H17	1910	4872	3325	4383	3822
H09	1894	4657	3855	4663	3920
H07	1886	4619	3989	4232	3964
H11	1861	4184	3881	4306	3668
H15	1822	4494	3929	4527	3885
H16	1812	4972	3774	4222	3772
H04	1787	4733	4023	4541	3825
H03	1723	4313	3756	4139	3733
H14	1693	4906	4027	4688	3870
H13	1657	4108	4086	4570	4047
H12	1620	4138	3856	4233	3776
H18	1574	4413	3848	4581	3994
H79	1503	3648	3379	3962	3284
H78	1378	3668	3686	4117	3297
H99	1030	2668	2044	3174	3063
Mean	1660	4230	3129	4047	3554
Repeatability	0.67	0.82	0.93	0.88	0.98
LSD	1087	1521	1165	1140	375
CV	27	15	27	21	25

## Discussion

When drought and *S. hermonthica* occur simultaneously in production fields, they inflict sever damage to growth and productivity of maize. Breeding maize in IITA for many year employed independent screening protocols to develop maize germplasm with either tolerance to drought (Campos et al., 2006: Cooper et al., 2014: Edmeades, 2013) or resistance to *S. hermonthica* (Kim, 1996; Kling et al., 2000), but not with tolerance to the combination of the two stresses. The present study was therefore conducted to examine the extent to which maize hybrids that simultaneously express tolerance to drought and resistance to *S. hermonthica* could be developed through sequential selection using reliable screening protocols established for the two stresses. The results of regional trials evaluated under MDS, WW, STRIN, and STRNO showed yield reductions of more than 60% under MDS for 4 years and 65% in a susceptible commercial hybrid under STRIN, indicating the adequacy of stress levels imposed during evaluation of hybrids for 5 years. Similarly, Bänziger et al. (2000) considers managed drought stress levels leading to 40 to 60% yield reductions appropriate to distinguish maize genotypes with tolerance genes. Moreover, the high repeatability estimates for grain yield recorded under MDS, WW, STRIN, and STRNO conditions demonstrated that the managements of both stressful and non-stressful growing conditions were adequate in eliciting consistent genetic differences among the DTSTR and benchmark hybrids across years and environments.

The success in breeding for tolerance to stress combinations was assessed in the present study by measuring improvement in grain yield of the DTSTR hybrids achieved under each stress condition relative to the yield potential of the commercial benchmark hybrids. The DTSTR hybrids produced higher average grain yields than the STR hybrids under MDS and WW and the non-DTSTR hybrids under MDS, WW, STRIN, and STRNO conditions, indicating that use of the sequential selection scheme can generate hybrids combining tolerance to drought with resistance to *S. hermonthica* without compromising yields under non-stressful growing conditions. These results are consistent with approaches advocated by other workers who promote the development of crop varieties with tolerance to multiple stresses that also have inherent capacity to maintain high rates of photosynthesis, growth rates, and yield under non-stressful production conditions (Condon et al., 2004; Morison et al., 2008; Bechtold et al., 2010). Such crop varieties allow farmers to attain high level of productivity in years when the occurrence of drought stress and *S. hermonthica* infestation are severe in their fields while optimizing harvested grains during favorable growing conditions.

In the current study, 60 to 71% of the DTSTR hybrids were high yielding and had greater adaptability across environments under each of MDS, WW, STRIN, and STRNO conditions. In contrast, most of the non-DTSTR hybrids produced low grain yields and were poorly adapted to environments particularly under stressful testing conditions. These findings signify the importance of fixing desirable stress-tolerant traits in parental lines through sequential selections using carefully controlled screening protocols to productivity increases expressed in hybrids under both parasite pressure and drought stress. In this selection scheme, the inbred lines were first selected under artificial *Striga* infestation based on less visible reduction in plant height, stem diameter, leaf chlorosis, leaf scorching, ear size, and tassel size and fewer emerged parasites counted from maize roots (Kim, 1994; Kling et al., 2000; Menkir et al., 2007). The resulting improvements in the overall plant growth and reductions in the number of emerged parasites may lead to reduced parasite damage on host photosynthesis (Graves et al., 1989; Gurney et al., 1995; Smith et al., 1995; Watling and Press, 2001). Gurney et al. (2002) found that a tolerant maize cultivar that produced high grain yield in the presence of *Striga asiatica* infection maintained higher rates of photosynthesis in the field in comparison to a susceptible cultivar. As drought stress reduces leaf area, accelerates leaf senescence, decreases leaf source activity, and changes numerous physiological and biochemical processes (Zaidi et al., 2004; Wahid et al., 2005; Li et al., 2018), the retention of green leaf area in the DTSTR hybrids under parasite infection may promote high rates of photosynthesis leading to increased kernel setting and grain yield under drought stress (Li et al., 2018; Tani et al., 2019). At the same time, the emphasis on enhanced vegetative growth, less visible leaf senescence, and synchronized male-female flowering of lines during the second stage of selection under managed drought stress may further accentuate photosynthetic activity to boost performance of the DTSTR hybrids under both drought stress and *Striga* infestation. Taylor et al. (1996) found that drought tolerant maize genotypes accumulate less ABA in their leaves after receiving the signal from the roots infected with *Striga* and their photosynthetic rate is therefore not reduced. Taking these findings together, pyramiding stress-tolerant traits through several cycles of sequential selection during inbreeding until homozygous lines are developed can increase the frequency of favorable dominant alleles for grain yield that confer superior performance in the DTSTR hybrids under both drought stress and parasite infection.

The significant phenotypic and modest positive genetic correlations between grain yields recorded under MDS and STRIN in our study suggest that yield was mediated mainly by the common set of genes that regulated similar responses in hybrids under both drought stress and parasite pressure (Lorenzana and Bernardo, 2008). Sun et al. (2015) identified some stress-responsive genes showing overlapping expression patterns in rice with pleiotropic effects on adaptive responses to drought stress and *S. hermonthica* infection. Several other studies also found many unique genes up-regulated under combined drought stress and plant pathogen infection (Narsai et al., 2013; Kissoudis et al., 2014; Rejeb et al., 2014; Pandey et al., 2015; Ramegowda and Senthil-Kumar, 2015), which could be involved in regulating common physiological and biochemical mechanisms that modulate defense responses of crop plants to multiple biotic and abiotic stresses (Kissoudis et al., 2014; Pandey et al., 2015; Ramegowda and Senthil-Kumar, 2015).

As the common maize production system in tropical Africa is rainfed, yield trials conducted across diverse field environments permit reliable assessment of the responses of hybrids to numerous factors occurring in production fields and identify adapted hybrids for cultivation. The high repeatability value observed in MET in our study suggest that trait expression was mainly determined by differences in the genetic makeup of the hybrids rather than by differences in precipitation, temperature, soil properties, incidence of diseases and pests, and crop management practices encountered during field testing. Most of the DTSTR hybrids evaluated in MET had favorable ranks across environments and produced higher mean grain yields in comparison to the STR and non-DTSTR hybrids. Nearly 38% of the DTSTR hybrids were found to be high yielding and had better adaptability under MDS and STRIN conditions as well as in MET. The observed significant phenotypic and modest genetic correlations of yield measured in MET with yields recorded under MDS, WW, STRIN, and STRNO conditions suggest that pyramiding of stress-tolerant traits and allelic combinations in parental lines through sequential selection can also impart a broad-spectrum of plasticity in hybrids allowing them to thrive and maintain consistently high grain yields in diverse field growing environments.

Half of the DTSTR hybrids evaluated in the current study simultaneously expressed tolerance to drought and resistance to *S. hermonthica*, highlighting that these traits were successfully combined to increase productivity in areas where they co-occur. The best seven DTSTR hybrids produced consistently higher grain yields than the STR commercial hybrids under MDS, WW, and in MET but were competitive to the latter under STRIN and STRNO conditions, indicating the potential that exists in achieving high grain yields across diverse production conditions possibly due to the absence of physiological tradeoffs between optimal and stressful environments. The best seven DTSTR hybrids can be recommended as potential candidates for further evaluation, registration, and release in tropical savannas where their predictable and consistent yields will be valuable. Furthermore, parents of the DTSTR hybrids offer breeders with unique traits and novel alleles to further improve maize inbred lines for developing new hybrids that combine much higher levels of resistance to *S. hermonthica* with tolerance to drought. We believe that the sequential selection scheme can help in the development of more stress resilient maize hybrids that sustain less damage in areas affected by the co-occurrence of drought stress and *S. hermonthica* infestation.

In summary, the sequential selection of inbred lines for desirable traits under *Striga* infestation and managed drought stress had frequently led to improvements in yield potential and adaptability of hybrids across stressful and non-stressful growing conditions. Among the 38 DTSTR hybrids that simultaneously expressing tolerance to drought with resistance to *S. hermonthica,* 14 yielded well in MET indicating that this selection scheme was effective in pyramiding stress-tolerant traits that are also beneficial across a range of rainfed field environments. Our study thus presents a clear case for breeders to use the sequential selection scheme to underpin productivity in areas affected by co-occurrence of recurrent drought and *Striga* infestation. Since little is known about the response of maize genotypes to the simultaneous presence of drought stress and *Striga* infestation that may not always be additive (Pandey et al., 2015; Ramegowda and Senthil-Kumar, 2015), the DTSTR hybrids and their parental lines can be suitable candidates for testing under combined stress conditions to unravel the physiological, biochemical, and molecular components contributing to productivity that can be targeted for breeding to develop more resilient maize cultivars with better adaptation to production conditions in farmers' fields. In addition, the DTSTR inbred lines can be used as elite gene pools for breeders to further improve resilience in hybrids for areas affected by the co-occurrence of drought stress and *S. hermonthica* infestation.

## Data Availability Statement

All yield data generated for this study are included in the article/**Supplementary Material**.

## Author Contributions

AM drafted the manuscript. JC conducted analyses and contributed to write-up of the different sections of the manuscript. SM coordinated conduct of regional trials and collection of data under managed drought stress and artificial *Striga* infestation. BB assisted in organizing the data for analyses and preparation of figures and tables. OM, PR, MC, A-MY, GO, and HA conducted regional trials in several locations in their respective countries, collected data and provided for analyses.

## Funding

This study was financed by the Drought Tolerant Maize for Africa and Stress Tolerant Maize for Africa Projects with funding from the Bill and Melinda Gates Foundation (BMGF).

## Conflict of Interest

The authors declare that the research was conducted in the absence of any commercial or financial relationships that could be construed as a potential conflict of interest.

## Abbreviations

DTSTR, Drought tolerant and *Striga* resistant; STR, *Striga* resistant commercial hybrids; non-DTSTR, Conventional commercial hybrids and a farmer preferred local maize variety; MDS, Managed drought stress; WW, Fully irrigated condition; STRIN, *Striga* infested condition; STRNO, *Striga* non-infested condition; MET, Multi-environment trial.
