# Supplementary figures and images for: Stacking Tolerance to Drought and Resistance to a Parasitic Weed in Tropical Hybrid Maize for Enhancing Resilience to Stress Combinations
Source: Front Plant Sci. 2020 Feb 28;11:166. doi: 10.3389/fpls.2020.00166 (PMC7061855; doi:10.3389/fpls.2020.00166)

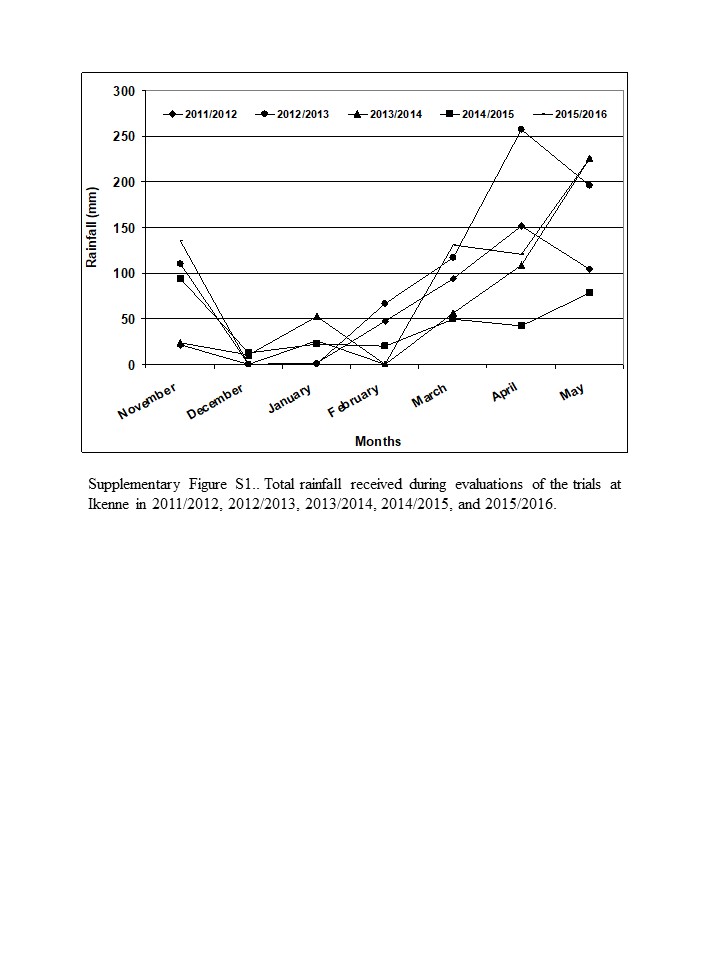

Supplement: Supplementary file 1 [file Image_1.jpeg]

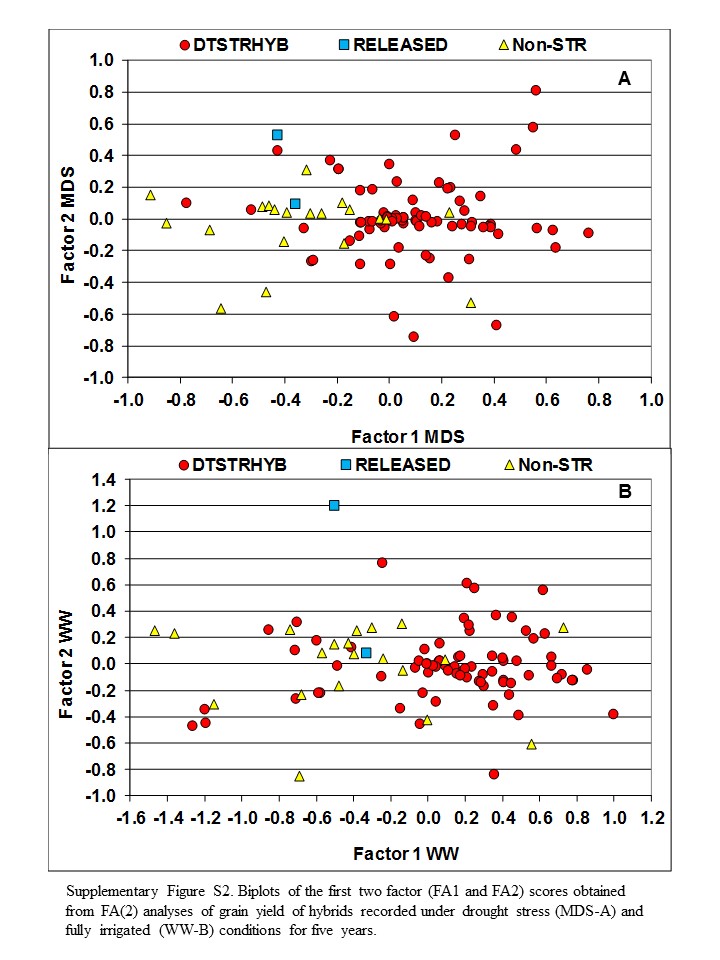

Supplement: Supplementary file 2 [file Image_2.jpeg]

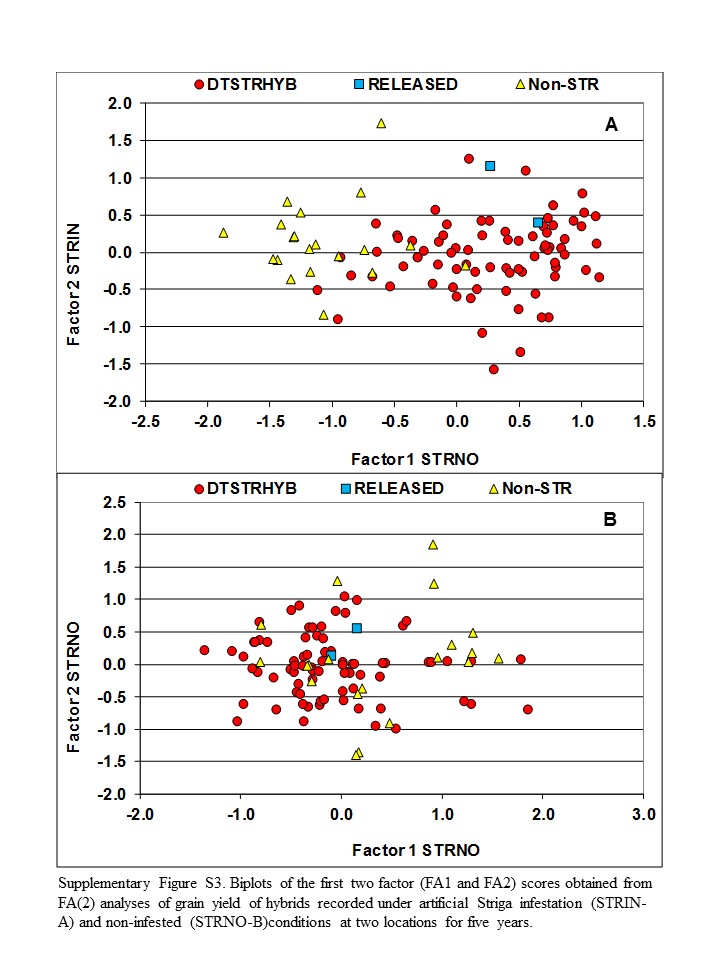

Supplement: Supplementary file 3 [file Image_3.jpeg]

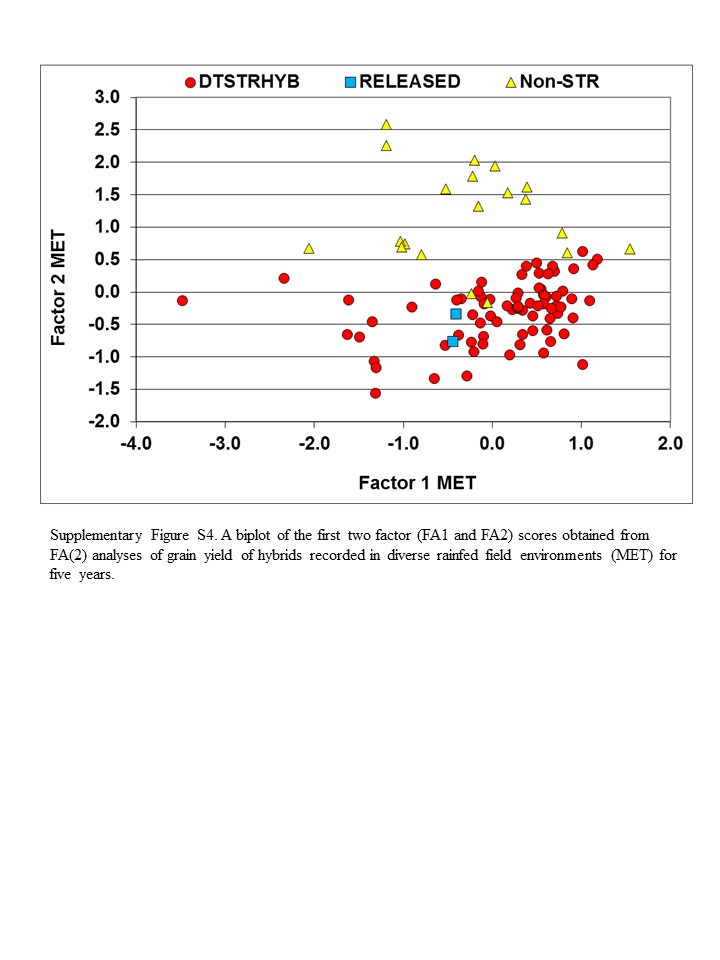

Supplement: Supplementary file 4 [file Image_4.jpeg]
