# Supplementary material for: Stacking Tolerance to Drought and Resistance to a Parasitic Weed in Tropical Hybrid Maize for Enhancing Resilience to Stress Combinations
Source: Front Plant Sci. 2020 Feb 28;11:166. doi: 10.3389/fpls.2020.00166 (PMC7061855; doi:10.3389/fpls.2020.00166)
Supplement: Supplementary file 5 [file Table_1.doc]

Supplementary Table S1. List of hybrids included in the regional trials evaluated under stressful and favourable growing conditions as well as in divers rainfed field environments for five years

| Hybrids | Pedigree |
| --- | --- |
| H01 | ACRSYN-W-S2-173-B*4/TZLCompIC4S1-37-5-BBB/IWD-SYN-STR-C3-47-1-BB |
| H02 | ZDiploBC4-19-4-1-#-3-1-B-1-B*4/TZLCompIC4S1-37-1-B*4/IWD-SYN-STR-C3-52-1-BB |
| H03 | ZDiploBC4-19-4-1-#-3-1-B-1-B*4/TZLCompIC4S1-37-1-B*4/IWD-SYN-STR-C3-53-2-BB |
| H04 | ACRSYN-W-S2-173-B*4/TZLCompIC4S1-37-1-B*4/IWD-SYN-STR-C3-53-2-BB |
| H05 | ACRSYN-W-S2-173-B*4/TZLCompIC4S1-37-1-B*4/IWD-SYN-STR-C3-55-3-BB |
| H06 | ACRSYN-W-S2-173-B*4/TZLCompIC4S1-37-5-BBB/IWD-SYN-STR-C3-55-3-BB |
| H07 | ZDiploBC4-472-2-2-1-2-3-B-1-B*5/TZLCompIC4S1-37-5-BBB/IWD-SYN-STR-C3-53-2-BB |
| H08 | ZdiploBC4-472-2-2-1-2-3-B*6/TZLCompIC4S1-37-5-BBB/IWD-SYN-STR-C3-55-3-BB |
| H09 | ZDiploBC4-19-4-1-#-3-1-B-1-B*4/TZLCompIC4S1-37-5-BBB/IWD-SYN-STR-C3-55-3-BB |
| H10 | ZDiploBC4-472-2-2-1-2-3-B-1-B*5/TZLCompIC4S1-37-5-BBB/IWD-SYN-STR-C3-67-1-BB |
| H11 | ACRSYN-W-S2-173-B*4/TZLCompIC4S1-37-5-BBB/IWD-SYN-STR-C3-32-2-BB |
| H12 | ZDiploBC4-19-4-1-#-3-1-B-1-B*4/TZLCompIC4S1-37-5-BBB/IWD-SYN-STR-C3-50-2-BB |
| H13 | ACRSYN-W-S2-173-B*4/TZLCompIC4S1-37-1-B*4/IWD-SYN-STR-C3-52-1-BB |
| H14 | ZdiploBC4-472-2-2-1-2-3-B*6/TZLCompIC4S1-37-5-BBB/IWD-SYN-STR-C3-52-4-BB |
| H15 | ACRSYN-W-S2-173-B*4/TZLCompIC4S1-37-1-B*4/IWD-SYN-STR-C3-52-3-BB |
| H16 | ZDiploBC4-472-2-2-1-2-3-B-1-B*5/TZLCompIC4S1-37-5-BBB/IWD-SYN-STR-C3-55-3-BB |
| H17 | ((Z. Diplo.BC4-472-2-2-1-2-3-B-1-B-B-B-B-B/Z.Diplo.BC4-19-4-1-#-3-1-B-1-B-B-B-B)-25-1-B-B/(ACRSYN-W-S2-173-B-B-B-B/TZL Comp. IC4 S1-37-1-B-B-B-B)-36-B-B-B-B)/IITATZISTR1133 |
| H18 | ((Z. Diplo.BC4-472-2-2-1-2-3-B-1-B-B-B-B-B/Z.Diplo.BC4-19-4-1-#-3-1-B-1-B-B-B-B)-43-1-B-B/(ACRSYN-W-S2-173-B-B-B-B/TZL Comp. IC4 S1-37-5-B-B-B)-3-B-B-B-B)/IITATZISTR1133 |
| H19 | (ZDiploBC4-472-2-2-1-2-3-B-1-B*5/ZDiploBC4-19-4-1-#-3-1-B-1-B*4)-25-1-BB/(ACRSYN-W-S2-173-B*4/TZLCompIC4S1-37-5-BBB)-38-1-1-BB/IITATZISTR1134 |
| H20 | (ZDiploBC4-472-2-2-1-2-3-B-1-B*5/ZDiploBC4-19-4-1-#-3-1-B-1-B*4)-2-1-BB/(ACRSYN-W-S2-173-B*4/TZLCompIC4S1-37-5-BBB)-4-B*4/IITATZISTR1134 |
| H21 | ZdiploBC4-472-2-2-1-2-3-B*6/(ACRSYN-W-S2-173-B*4/TZLCompIC4S1-37-1-B*4)-57-B/IWD-SYN-STR-C3--70-2-B |
| H22 | ZdiploBC4-472-2-2-1-2-3-B*6/(ACRSYN-W-S2-173-B*4/TZLCompIC4S1-37-5-BBB)-4-B/IWD-SYN-STR-C3--70-2-B |
| H23 | ZeaDiploBC4-WC3-29-3-1-B*4/(ACRSYN-W-S2-173-B*4/TZLCompIC4S1-37-5-BBB)-27-B/IWD-SYN-STR-C3--32-2-BB |
| H24 | (ZDiploBC4-472-2-2-1-2-3-B-1-B*5/ZDiploBC4-19-4-1-#-3-1-B-1-B*4)-25-1-BB/(ACRSYN-W-S2-173-B*4/TZLCompIC4S1-37-5-BBB)-56-B*4/IWD-SYN-STR-C3--52-1-B*5 |
| H25 | ZDiploBC4-19-4-1-#-3-1-B-1-B*4/(ACRSYN-W-S2-173-B*4/TZLCompIC4S1-37-1-B*4)-16-B/(IITATZI1872 |
| H26 | (1393/Z.Diplo.BC4-19-4-1-#-3-1-B-1-B*4)-46-B-B-B-B/(ACRSYN-W-S2-173-B-B-B-B/TZL Comp. IC4 S1-37-5-B-B-B)-31-1-1-B-B/IITATZISTR1129 |
| H27 | ACRSYN-W-S2-173-B*6/TZLCompIC4S1-37-5-BBB/TZISTR1128 |
| H28 | ZdiploBC4-472-2-2-1-2-3-B*8/TZLCompIC4S1-37-5-B*5/TZISTR1128 |
| H29 | ACRSYN-W-S2-173-B*6/TZLCompIC4S1-37-5-BBB/TZISTR1132 |
| H30 | ZdiploBC4-472-2-2-1-2-3-B*6/(ACRSYN-W-S2-173-B*4/TZLCompIC4S1-37-5-BBB)-4-B/(IITATZI1872 |
| H31 | ZeADiploBC4-WC3-29-3-1-B*4/(ACRSYN-W-S2-173-B*4/TZLCompIC4S1-37-5-BBB)-27-B/IITATZISTR1129 |
| H32 | ZdiploBC4-376-1-1-#-3-1-B-2-BBB/ACR97TZL-CCOMP1-Y-S3-34-3-BBB/ACR97SYN-Y-S1-76-B-B-B-B |
| H33 | ACRSYN-W-S2-173-B*4/TZLCompIC4S1-37-1-B*4/IWD-SYN-STR-C3-32-2-BB |
| H34 | ZDiploBC4-19-4-1-#-3-1-B-1-B*4/TZLCompIC4S1-37-1-B*4/IWD-SYN-STR-C3-67-1-BB |
| H35 | ACRSYN-W-S2-173-B*4/TZLCompIC4S1-37-1-B*4/IWD-SYN-STR-C3-67-1-BB |
| H36 | ACRSYN-W-S2-173-B*4/TZLCompIC4S1-37-5-BBB/IWD-SYN-STR-C3-67-1-BB |
| H37 | ZDiploBC4-472-2-2-1-2-3-B-1-B*5/TZLCompIC4S1-37-5-BBB/IWD-SYN-STR-C3-32-2-BB |
| H38 | ACRSYN-W-S2-173-B*4/TZLCompIC4S1-37-5-BBB/IWD-SYN-STR-C3-52-4-BB |
| H39 | ACRSYN-W-S2-173-B*4/TZLCompIC4S1-37-5-BBB/IWD-SYN-STR-C3-53-2-BB |
| H40 | ZDiploBC4-472-2-2-1-2-3-B-1-B*5/TZLCompIC4S1-37-1-B*4/IWD-SYN-STR-C3-47-1-BB |
| H41 | ZDiploBC4-472-2-2-1-2-3-B-1-B*5/TZLCompIC4S1-37-1-B*4/IWD-SYN-STR-C3-50-2-BB |
| H42 | ACRSYN-W-S2-173-B*4/TZLCompIC4S1-37-1-B*4/IWD-SYN-STR-C3-52-4-BB |
| H43 | ZDiploBC4-19-4-1-#-3-1-B-1-B*4/ACRSYN-W-S2-173-B*4/IWD-SYN-STR-C3-52-1-B*5 |
| H44 | ZdiploBC4-472-2-3-4-3-B-2-B*5/TZLCompIC4S1-37-5-BBB/IWD-SYN-STR-C3-70-1-B*5 |
| H45 | ACRSYN-W-S2-173-B*4/TZLCompIC4S1-37-5-BBB/IWD-SYN-STR-C3-52-4-BB |
| H46 | ZdiploBC4-472-2-2-1-2-3-B*6/TZLCompIC4S1-37-1-B*4/IWD-SYN-STR-C3-70-1-BB |
| H47 | ZDiploBC4-472-2-2-1-2-3-B-1-B*5/TZLCompIC4S1-37-1-B*4/IWD-SYN-STR-C3-70-1-BB |
| H48 | ZDiploBC4-19-4-1-#-3-1-B-1-B*4/TZLCompIC4S1-37-5-BBB/IWD-SYN-STR-C3-32-2-BB |
| H49 | ((1393/Z.Diplo.BC4-19-4-1-#-3-1-B-1-B*4)-61-1-1-B-B/(ACRSYN-W-S2-173-B-B-B-B/TZL Comp. IC4 S1-37-1-B-B-B-B)-54-B-B-B-B))/IITATZISTR1117 |
| H50 | ((Z. Diplo.BC4-472-2-2-1-2-3-B-1-B-B-B-B-B/Z.Diplo.BC4-19-4-1-#-3-1-B-1-B-B-B-B)-2-1-B-B/(ACRSYN-W-S2-173-B-B-B-B/TZL Comp. IC4 S1-37-5-B-B-B)-4-B-B-B-B)/IITATZISTR1134 |
| H51 | ((Z. Diplo.BC4-472-2-2-1-2-3-B-1-B-B-B-B-B/Z.Diplo.BC4-19-4-1-#-3-1-B-1-B-B-B-B)-25-1-B-B/(ACRSYN-W-S2-173-B-B-B-B/TZL Comp. IC4 S1-37-5-B-B-B)-38-1-1-B-B)/IITATZISTR1134 |
| H52 | (1393/ZDiploBC4-19-4-1-#-3-1-B-1-B*4)-61-1-1-BB/(ACRSYN-W-S2-173-B*4/TZLCompIC4S1-37-1-B*4)-54-B*4/IITATZISTR1117 |
| H53 | ZDiploBC4-472-2-2-1-2-3-B-1-B*5/(ACRSYN-W-S2-173-B*4/TZLCompIC4S1-37-5-BBB)-35-B/IWD-SYN-STR-C3-32-2-BB |
| H54 | (1393/ZDiploBC4-19-4-1-#-3-1-B-1-B*4)-12-B*4/(ACRSYN-W-S2-173-B*4/TZLCompIC4S1-37-5-BBB)-17-B*4/IWD-SYN-STR-C3-1-1-B*5 |
| H55 | (ZDiploBC4-472-2-2-1-2-3-B-1-B*5/ZDiploBC4-19-4-1-#-3-1-B-1-B*4)-26-1-BB/(ACRSYN-W-S2-173-B*4/TZLCompIC4S1-37-1-B*4)-50-B*4/IWD-SYN-STR-C3-47-1-B*5 |
| H56 | (ZDiploBC4-472-2-2-1-2-3-B-1-B*5/ZDiploBC4-19-4-1-#-3-1-B-1-B*4)-2-1-BB/(ACRSYN-W-S2-173-B*4/TZLCompIC4S1-37-5-BBB)-4-B*4/IWD-SYN-STR-C3-52-1-B*5 |
| H57 | (ZDiploBC4-472-2-2-1-2-3-B-1-B*5/ZDiploBC4-19-4-1-#-3-1-B-1-B*4)-26-1-BB/(ACRSYN-W-S2-173-B*4/TZLCompIC4S1-37-5-BBB)-40-B*4/IWD-SYN-STR-C3-52-1-B*5 |
| H58 | ZDiploBC4-19-4-1-#-3-1-B-1-B*6/TZLCompIC4S1-37-5-B*5/IWD-SYN-STR-C3-32-2-BB |
| H59 | ZdiploBC4-472-2-2-1-2-3-B*8/TZLCompIC4S1-37-5-B*5/IWD-SYN-STR-C3-32-2-BB |
| H60 | (ZDiploBC4-472-2-2-1-2-3-B-1-B*5/ZDiploBC4-19-4-1-#-3-1-B-1-B*4)-2-1-BB/(ACRSYN-W-S2-173-B*4/TZLCompIC4S1-37-5-BBB)-4-B*4/IITATZISTR1134 |
| H61 | 4001xB73LPAx4001-6-2-3-BBB/ACR97TZL-CCOMP1-Y-S3-24-1-B*4 |
| H62 | ZdiploBC4-376-1-1-#-3-1-B-2-BBB/ACR97TZL-CCOMP1-Y-S3-34-3-BBB/ACR97SYN-Y-S1-76-B*4 |
| H63 | (1393/ZDiploBC4-19-4-1-#-3-1-B-1-B*4)-40-BB/IWD-SYN-STR-C3-50-2-BBB/TZLCompIC4S1-37-1-B*6 |
| H64 | ZDiploBC4-19-4-1-#-3-1-B-1-B*4/(ACRSYN-W-S2-173-B*4/TZLCompIC4S1-37-1-B*4)-16-B/IWD-SYN-STR-C3-70-2-B |
| H65 | ZDiploBC4-19-4-1-#-3-1-B-1-B*4/(ACRSYN-W-S2-173-B*4/TZLCompIC4S1-37-1-B*4)-32-B/IWD-SYN-STR-C3-70-2-B |
| H66 | STRLowEmergPoolCOS3670-1-5-3-3-B-1-B*7/(ACRSYN-W-S2-173-B*4/TZLCompIC4S1-37-5-BBB)-3-B/IWD-SYN-STR-C3-70-2-B |
| H67 | Acr.Syn-W S2-173-B-B-B/Z.Diplo BC4-472-2-3-4-3-B-2-B/TZLCompIC4S1-37-1-B*6 |
| H68 | TZISTR1008/TZISTR1007/IWD-SYN-STR-C3-50-2-B-B |
| H69 | 0601-6STR/IWD-SYN-STR-C3-52-4-B*5 |
| H70 | TZISTR1199/TZISTR1271/IWD-SYN-STR-C3-67-1-B*5 |
| H71 | Acr.Syn-W S2-173-B-B-B/Z.Diplo BC4-472-2-3-4-3-B-2-B/TZLCompIC4S1-37-5-B*6 |
| H72 | Z.diplo.BC4-472-2-2-1-2-3-B-B-B-B-B-B/(ACRSYN-W-S2-173-B-B-B-B/TZL Comp. IC4 S1-37-5-B-B-B)-4-B/IWD-SYN-STR-C3-70-2-B |
| H73 | (Z. Diplo.BC4-472-2-2-1-2-3-B-1-B-B-B-B-B/Z.Diplo.BC4-19-4-1-#-3-1-B-1-B-B-B-B)-2-1-B-B/(ACRSYN-W-S2-173-B-B-B-B/TZL Comp. IC4 S1-37-5-B-B-B)-4-B-B-B-B/IWD-SYN-STR-C3--52-1-B-B |
| H74 | (Z. Diplo.BC4-472-2-2-1-2-3-B-1-B-B-B-B-B/Z.Diplo.BC4-19-4-1-#-3-1-B-1-B-B-B-B)-25-1-B-B/(ACRSYN-W-S2-173-B-B-B-B/TZL Comp. IC4 S1-37-5-B-B-B)-56-B-B-B-B/IWD-SYN-STR-C3-52-1-B-B |
| H75 | ZDiploBC4-472-2-2-1-2-3-B-1-B*7/TZLCompIC4S1-37-1-B*6/IWD-SYN-STR-C3-67-1-B*5 |
| H76 | ZdiploBC4-472-2-2-1-2-3-B*8/TZLCompIC4S1-37-5-B*5/IWD-SYN-STR-C3-67-1-B*5 |
| H77 | ACRSYN-W-S2-173-B-B-B-B-B-B/TZLCompIC4S1-37-5-B*5/IWD-SYN-STR-C3-67-1-B*5 |
| H78 | RSTRHYB01 |
| H79 | RSTRHYB02 |
| H80 | COMHYB01 |
| H81 | COMHYB02 |
| H82 | COMHYB03 |
| H83 | COMHYB04 |
| H84 | COMHYB05 |
| H85 | COMHYB06 |
| H86 | COMHYB07 |
| H87 | COMHYB08 |
| H88 | COMHYB09 |
| H89 | COMHYB10 |
| H90 | COMHYB11 |
| H91 | COMHYB12 |
| H92 | COMHYB13 |
| H93 | COMHYB14 |
| H94 | COMHYB15 |
| H95 | COMHYB16 |
| H96 | COMHYB17 |
| H97 | COMHYB18 |
| H98 | COMHYB19 |
| H99 | Local check |

Supplementary Table S2. Average monthly minimum and maximum temperatures for each year during testing of the hybrids under *Striga* infested and non-infested conditions.

|  |  | Average monthly temperature ^0^C | | | | | | |
| --- | --- | --- | --- | --- | --- | --- | --- | --- |
| Test year | Range | May | June | July | August | September | October | November |
|  |  |  |  |  | Kubwa |  |  |  |
| 2012 | Minimum | 23 | 22 | 21 | 21 | 21 | 22 | 19 |
|  | Maximum | 30 | 28 | 26 | 27 | 27 | 29 | 30 |
| 2013 | Minimum | 23 | 22 | 21 | 21 | 21 | 21 | 21 |
|  | Maximum | 30 | 29 | 28 | 29 | 29 | 31 | 34 |
| 2014 | Minimum | 24 | 22 | 21 | 21 | 21 | 21 | 20 |
|  | Maximum | 32 | 30 | 29 | 27 | 28 | 30 | 32 |
| 2015 | Minimum | 24 | 23 | 22 | 22 | 22 | 22 | 19 |
|  | Maximum | 34 | 31 | 28 | 28 | 29 | 30 | 33 |
| 2016 | Minimum | 23 | 22 | 22 | 22 | 22 | 21 | 19 |
|  | Maximum | 31 | 29 | 28 | 28 | 28 | 30 | 32 |
|  |  |  |  |  | Mokwa |  |  |  |
| 2012 | Minimum | 23 | 22 | 21 | 21 | 21 | 22 | 20 |
|  | Maximum | 33 | 31 | 29 | 29 | 30 | 31 | 34 |
| 2013 | Minimum | 23 | 22 | 22 | 21 | 21 | 22 | 20 |
|  | Maximum | 33 | 31 | 29 | 29 | 30 | 31 | 34 |
| 2014 | Minimum | 23 | 22 | 22 | 21 | 21 | 22 | 20 |
|  | Maximum | 33 | 31 | 29 | 29 | 29 | 31 | 34 |
| 2015 | Minimum | 23 | 22 | 22 | 21 | 21 | 22 | 20 |
|  | Maximum | 33 | 31 | 29 | 29 | 30 | 31 | 34 |
| 2016 | Minimum | 23 | 22 | 21 | 21 | 22 | 22 | 20 |
|  | Maximum | 33 | 31 | 29 | 29 | 30 | 31 | 33 |

Supplementary Table S3. Grain yield BLUP estimates of hybrids included in regional trials obtained under managed drought stress and fully irrigated conditions, artificial *Striga* infestation and non-infested conditions as well as in multiple rainfed field environments for five years

|  | Grain yield (kg/ha) | | | | |
| --- | --- | --- | --- | --- | --- |
| Hybrid | Managed drought stress | Fully irrigated | *Striga* infested | *Striga* non-infested | Multiple rainfed field environments |
| H01 | 2068 | 4578 | 3746 | 4374 | 3857 |
| H02 | 1644 | 3673 | 3529 | 3807 | 3573 |
| H03 | 1723 | 4313 | 3756 | 4139 | 3733 |
| H04 | 1787 | 4733 | 4023 | 4541 | 3825 |
| H05 | 1595 | 3555 | 3828 | 4143 | 3197 |
| H06 | 1988 | 4682 | 4390 | 4932 | 4031 |
| H07 | 1886 | 4619 | 3989 | 4232 | 3964 |
| H08 | 1850 | 4572 | 3770 | 4322 | 3636 |
| H09 | 1894 | 4657 | 3855 | 4663 | 3920 |
| H10 | 1748 | 4633 | 3857 | 4259 | 4023 |
| H11 | 1861 | 4184 | 3881 | 4306 | 3668 |
| H12 | 1620 | 4138 | 3856 | 4233 | 3776 |
| H13 | 1657 | 4108 | 4086 | 4570 | 4047 |
| H14 | 1693 | 4906 | 4027 | 4688 | 3870 |
| H15 | 1822 | 4494 | 3929 | 4527 | 3885 |
| H16 | 1812 | 4972 | 3774 | 4222 | 3772 |
| H17 | 1910 | 4872 | 3325 | 4383 | 3822 |
| H18 | 1574 | 4413 | 3848 | 4581 | 3994 |
| H19 | 1366 | 4220 | 3889 | 4433 | 3934 |
| H20 | 1769 | 4783 | 4159 | 4632 | 4173 |
| H21 | 1688 | 4489 | 3813 | 4328 | 3761 |
| H22 | 1819 | 4185 | 3315 | 3916 | 3162 |
| H23 | 1667 | 4480 | 3218 | 4092 | 3464 |
| H24 | 1714 | 4223 | 3961 | 4806 | 3727 |
| H25 | 1505 | 3704 | 2420 | 3112 | 2803 |
| H26 | 1668 | 4574 | 2477 | 4273 | 3489 |
| H27 | 1648 | 4567 | 3835 | 4797 | 3608 |
| H28 | 1685 | 4597 | 3824 | 4484 | 3682 |
| H29 | 1776 | 4207 | 4160 | 4367 | 3953 |
| H30 | 1508 | 3868 | 2972 | 3757 | 3277 |
| H31 | 1640 | 4382 | 3123 | 4027 | 3439 |
| H32 | 1597 | 3483 | 2864 | 3163 | 2150 |
| H33 | 1783 | 4621 | 3255 | 4220 | 3646 |
| H34 | 1899 | 4127 | 3596 | 3861 | 3764 |
| H35 | 1998 | 5233 | 2866 | 4409 | 3832 |
| H36 | 1824 | 5226 | 3160 | 4323 | 3888 |
| H37 | 1076 | 2878 | 2314 | 2817 | 2067 |
| H38 | 1880 | 4568 | 3011 | 3885 | 3877 |
| H39 | 1700 | 4674 | 3526 | 4004 | 3738 |
| H40 | 1938 | 4786 | 3216 | 4235 | 3527 |
| H41 | 1738 | 4384 | 3681 | 4330 | 3674 |
| H42 | 1698 | 4479 | 3047 | 4313 | 3689 |
| H43 | 1790 | 4338 | 3141 | 3889 | 3722 |
| H44 | 1777 | 4405 | 2817 | 3451 | 3159 |
| H45 | 1673 | 5119 | 3838 | 3977 | 3855 |
| H46 | 1409 | 3291 | 2754 | 3557 | 2684 |
| H47 | 1861 | 4237 | 3080 | 4216 | 3563 |
| H48 | 1936 | 4918 | 3415 | 3935 | 3555 |
| H49 | 1892 | 4365 | 1973 | 3915 | 3550 |
| H50 | 1650 | 4277 | 3028 | 4263 | 4074 |
| H51 | 1760 | 4692 | 3349 | 4144 | 3630 |
| H52 | 1723 | 4344 | 2146 | 3904 | 3436 |
| H53 | 1622 | 4161 | 3666 | 4335 | 3193 |
| H54 | 1572 | 4025 | 2277 | 3626 | 3278 |
| H55 | 1585 | 4768 | 3902 | 4377 | 3998 |
| H56 | 1744 | 4779 | 4018 | 4639 | 4030 |
| H57 | 1536 | 3325 | 2284 | 2716 | 2642 |
| H58 | 1722 | 4260 | 2597 | 3781 | 3286 |
| H59 | 1293 | 3271 | 2850 | 3261 | 2492 |
| H60 | 1693 | 3784 | 2287 | 3016 | 2702 |
| H61 | 1537 | 3776 | 2800 | 3421 | 2449 |
| H62 | 1586 | 3686 | 2608 | 3377 | 2290 |
| H63 | 1666 | 4412 | 3783 | 4004 | 3508 |
| H64 | 1799 | 4682 | 2792 | 3992 | 3565 |
| H65 | 1805 | 4555 | 3619 | 4222 | 3945 |
| H66 | 1673 | 4261 | 3352 | 3928 | 3502 |
| H67 | 1515 | 4516 | 3524 | 4275 | 3785 |
| H68 | 1975 | 4601 | 4000 | 4509 | 4133 |
| H69 | 1780 | 4230 | 3142 | 4138 | 4263 |
| H70 | 1657 | 4291 | 3467 | 4830 | 4032 |
| H71 | 1516 | 4442 | 3339 | 4607 | 3609 |
| H72 | 1579 | 4470 | 4185 | 4622 | 3896 |
| H73 | 1658 | 4269 | 3638 | 4576 | 3979 |
| H74 | 1670 | 4167 | 3903 | 4320 | 3838 |
| H75 | 1490 | 4225 | 3575 | 4383 | 4007 |
| H76 | 1771 | 4619 | 3555 | 4290 | 4215 |
| H77 | 1835 | 4378 | 3251 | 4568 | 3823 |
| H78 | 1378 | 3668 | 3686 | 4117 | 3297 |
| H79 | 1503 | 3648 | 3379 | 3962 | 3284 |
| H87 | 1918 | 4757 | 2158 | 4306 | 4111 |
| H88 | 1629 | 3842 | 1676 | 2877 | 2530 |
| H89 | 1481 | 4123 | 3186 | 4633 | 4206 |
| H80 | 1354 | 3969 | 1745 | 3375 | 3512 |
| H81 | 1115 | 3339 | 2055 | 3110 | 2758 |
| H82 | 1324 | 2974 | 1597 | 3202 | 3298 |
| H83 | 1878 | 4984 | 2379 | 3917 | 3736 |
| H84 | 1458 | 4332 | 2483 | 4549 | 4330 |
| H85 | 1448 | 3796 | 1832 | 4366 | 4019 |
| H86 | 1422 | 3742 | 1161 | 3319 | 3703 |
| H90 | 1649 | 4283 | 2517 | 4291 | 3911 |
| H91 | 1471 | 3904 | 2117 | 3522 | 3070 |
| H92 | 1378 | 3535 | 1928 | 3437 | 2801 |
| H93 | 1569 | 3786 | 1955 | 4122 | 3338 |
| H94 | 1512 | 3479 | 2693 | 3801 | 3342 |
| H95 | 1779 | 3706 | 2523 | 3775 | 3596 |
| H96 | 1695 | 3752 | 1856 | 3574 | 3269 |
| H97 | 1543 | 4005 | 1892 | 3516 | 3659 |
| H98 | 1363 | 3681 | 1774 | 3102 | 2371 |
| H99 | 1030 | 2668 | 2044 | 3174 | 3063 |
| Mean | 1660 | 4230 | 3129 | 4047 | 3554 |
| Repeatability | 0.70 | 0.82 | 0.93 | 0.88 | 0.98 |
| LSD (0.5) | 1066 | 1521 | 1165 | 1140 | 375 |
| CV | 32 | 15 | 27 | 21 | 25 |

Supplementary Table S4. Differences between hybrid groups (*P*-values) for yield BLUPs under managed drought stress and full irrigation at Ikenne during the dry season across five years.

| Groups | DTSTR hybrids | STR commercial hybrids | Non-DTSTR commercial hybrids |
| --- | --- | --- | --- |
|  | Drought stress | | |
| DTSTR hybrids |  | 0.0384 | <.0001 |
| STR commercial hybrids | 0.0384 |  | 0.6479 |
| Non-DTSTR commercial hybrids | <.0001 | 0.6479 |  |
|  | Fully irrigated | | |
| DTSTR hybrids |  | 0.0406 | <.0001 |
| STR commercial hybrids | 0.0406 |  | 0.6126 |
| Non-DTSTR commercial hybrids | <.0001 | 0.6126 |  |

Supplementary Table S5. FA1 and FA2 scores obtained from FA(2) analyses for hybrids included in regional trials and evaluated under managed drought stress and fully irrigated conditions, artificial *Striga* infestation and non-infested conditions as well as in multiple rainfed field environments for five years.

|  | Managed drought stress | | Fully irrigated | | *Striga* infested | | *Striga* non-infested | | Multiple rainfed field environments | |
| --- | --- | --- | --- | --- | --- | --- | --- | --- | --- | --- |
| Hybrid | FA1 | FA2 | FA1 | FA2 | FA1 | FA2 | FA1 | FA2 | FA1 | FA2 |
| H01 | 0.64 | -0.18 | 0.35 | -0.31 | 0.69 | 0.36 | -0.50 | 0.83 | 0.55 | 0.05 |
| H02 | -0.07 | 0.19 | -0.49 | -0.01 | 0.40 | -0.52 | 0.17 | -0.69 | -0.13 | -0.48 |
| H03 | 0.02 | -0.61 | 0.09 | -0.01 | 0.63 | -0.56 | -0.10 | 0.21 | 0.34 | -0.28 |
| H04 | 0.25 | 0.53 | 0.49 | -0.38 | 1.00 | 0.35 | -0.83 | -0.12 | 0.53 | 0.07 |
| H05 | 0.09 | -0.74 | -0.71 | 0.32 | 0.61 | 0.21 | 0.15 | 0.99 | -0.65 | -1.33 |
| H06 | 0.49 | 0.44 | 0.44 | -0.24 | 1.14 | -0.34 | -1.37 | 0.21 | 0.92 | 0.36 |
| H07 | 0.55 | 0.58 | 0.48 | 0.02 | 0.74 | -0.87 | -0.36 | 0.41 | 0.80 | 0.02 |
| H08 | 0.42 | -0.09 | 0.20 | 0.35 | 0.50 | -0.77 | -0.06 | 0.82 | 0.20 | -0.97 |
| H09 | 0.39 | -0.03 | 0.35 | 0.06 | 0.75 | 0.07 | -0.97 | 0.12 | 0.60 | -0.07 |
| H10 | 0.23 | 0.20 | 0.35 | -0.83 | 0.62 | -0.06 | 0.02 | 1.04 | 0.73 | -0.33 |
| H11 | 0.35 | 0.14 | 0.21 | 0.61 | 0.72 | 0.27 | -0.43 | 0.91 | 0.22 | -0.27 |
| H12 | -0.02 | -0.05 | -0.02 | 0.12 | 0.77 | 0.36 | -0.38 | 0.12 | 0.42 | -0.17 |
| H13 | 0.00 | -0.28 | -0.04 | -0.45 | 0.93 | 0.42 | -0.88 | -0.06 | 0.81 | -0.64 |
| H14 | 0.03 | 0.24 | 0.66 | 0.05 | 1.01 | 0.79 | -1.04 | -0.88 | 0.58 | -0.04 |
| H15 | 0.31 | -0.25 | 0.23 | 0.26 | 0.84 | 0.06 | -0.82 | 0.38 | 0.58 | -0.05 |
| H16 | 0.27 | 0.12 | 0.85 | -0.04 | 0.55 | 1.09 | -0.22 | -0.62 | 0.33 | 0.27 |
| H17 | 0.56 | -0.06 | 0.72 | -0.08 | 0.10 | 1.25 | -0.47 | 0.05 | 0.46 | -0.37 |
| H18 | -0.08 | -0.06 | 0.34 | -0.05 | 0.70 | 0.06 | -0.82 | 0.66 | 0.72 | -0.21 |
| H19 | -0.43 | 0.43 | 0.00 | -0.06 | 0.73 | 0.46 | -0.51 | -0.07 | 0.72 | -0.07 |
| H20 | 0.56 | 0.81 | 1.00 | -0.38 | 1.04 | -0.24 | -0.87 | 0.35 | 1.10 | -0.13 |
| H21 | 0.10 | 0.04 | 0.41 | 0.02 | 0.52 | -0.26 | -0.21 | -0.57 | 0.65 | -0.76 |
| H22 | 0.22 | 0.20 | -0.24 | 0.76 | 0.15 | -0.26 | 0.33 | -0.95 | -0.23 | -0.77 |
| H23 | 0.00 | 0.35 | 0.29 | -0.08 | 0.08 | -0.17 | 0.02 | -0.55 | -0.22 | -0.34 |
| H24 | 0.19 | 0.23 | 0.06 | 0.16 | 0.87 | 0.18 | -1.09 | 0.21 | 0.61 | -0.59 |
| H25 | -0.30 | -0.26 | -0.60 | 0.18 | -0.65 | 0.38 | 1.29 | -0.61 | -0.90 | -0.23 |
| H26 | 0.02 | 0.02 | 0.41 | -0.12 | -0.64 | 0.01 | -0.34 | 0.15 | -0.03 | -0.11 |
| H27 | -0.02 | 0.04 | 0.41 | -0.13 | 0.77 | 0.63 | -0.98 | -0.61 | 0.28 | -0.25 |
| H28 | 0.04 | -0.18 | 0.45 | -0.15 | 0.73 | 0.03 | -0.74 | 0.34 | 0.29 | -0.01 |
| H29 | 0.29 | 0.05 | 0.24 | -0.02 | 1.12 | 0.49 | -0.47 | -0.11 | 0.77 | -0.23 |
| H30 | -0.29 | -0.26 | -0.41 | 0.12 | -0.15 | 0.14 | 0.38 | -0.18 | -0.35 | -0.10 |
| H31 | -0.03 | -0.03 | 0.19 | -0.06 | -0.01 | 0.05 | 0.01 | 0.03 | -0.13 | -0.08 |
| H32 | -0.12 | -0.10 | -0.85 | 0.26 | -0.26 | 0.02 | 1.22 | -0.57 | -1.63 | -0.65 |
| H33 | 0.32 | -0.02 | 0.30 | -0.17 | 0.16 | -0.49 | 0.04 | 0.80 | 0.38 | 0.40 |
| H34 | 0.31 | -0.05 | -0.15 | -0.34 | 0.68 | -0.88 | 0.60 | 0.59 | 0.29 | -0.24 |
| H35 | 0.76 | -0.08 | 0.78 | -0.12 | -0.36 | 0.16 | -0.33 | 0.57 | -0.29 | -1.30 |
| H36 | 0.38 | -0.04 | 0.78 | -0.12 | 0.26 | -0.20 | -0.29 | 0.56 | 0.46 | -0.59 |
| H37 | -0.78 | 0.10 | -1.20 | -0.34 | -0.96 | -0.89 | 1.85 | -0.70 | -3.48 | -0.13 |
| H38 | 0.24 | -0.04 | 0.37 | 0.37 | -0.17 | 0.57 | 0.12 | -0.37 | 0.58 | -0.18 |
| H39 | 0.10 | -0.01 | 0.45 | 0.35 | 0.40 | -0.21 | -0.38 | -0.88 | 0.52 | 0.30 |
| H40 | 0.39 | -0.05 | 0.53 | 0.25 | 0.26 | 0.42 | -0.47 | -0.02 | -0.09 | -0.18 |
| H41 | 0.18 | -0.01 | 0.25 | 0.57 | 0.51 | -1.33 | -0.20 | 0.59 | -0.10 | -0.69 |
| H42 | 0.10 | -0.01 | 0.20 | -0.03 | -0.05 | -0.01 | -0.25 | 0.45 | 0.27 | -0.10 |
| H43 | 0.05 | -0.03 | 0.04 | -0.28 | -0.03 | -0.47 | 0.65 | 0.66 | 0.29 | -0.22 |
| H44 | 0.28 | -0.03 | 0.15 | -0.02 | -0.48 | 0.23 | 0.53 | -1.00 | -1.35 | -0.46 |
| H45 | 0.05 | -0.01 | 0.70 | -0.11 | 0.79 | -0.32 | 0.06 | -0.12 | 0.31 | -0.81 |
| H46 | -0.53 | 0.06 | -0.71 | 0.11 | -0.47 | 0.20 | 0.39 | -0.67 | -2.34 | 0.21 |
| H47 | 0.12 | -0.04 | -0.03 | -0.22 | 0.00 | -0.60 | -0.18 | 0.41 | -0.38 | -0.66 |
| H48 | 0.62 | -0.07 | 0.54 | -0.08 | 0.09 | 0.04 | 0.08 | -0.13 | -0.15 | 0.01 |
| H49 | 0.36 | -0.05 | 0.17 | 0.06 | -1.12 | -0.50 | 0.19 | -0.17 | -0.53 | -0.82 |
| H50 | -0.01 | 0.00 | 0.06 | 0.02 | -0.11 | 0.23 | -0.30 | -0.04 | 1.18 | 0.51 |
| H51 | 0.16 | -0.02 | 0.57 | 0.20 | 0.20 | 0.43 | -0.17 | 0.20 | 0.16 | -0.21 |
| H52 | 0.05 | 0.01 | 0.18 | 0.07 | -0.85 | -0.31 | 0.11 | 0.00 | -0.40 | -0.12 |
| H53 | -0.03 | -0.01 | -0.07 | -0.03 | 0.39 | 0.28 | -0.39 | -0.02 | -0.21 | -0.92 |
| H54 | -0.08 | -0.01 | -0.25 | -0.09 | -0.94 | -0.06 | 0.43 | 0.02 | -0.11 | -0.80 |
| H55 | -0.15 | -0.13 | 0.62 | 0.56 | 0.79 | -0.20 | -0.68 | -0.21 | 0.50 | 0.45 |
| H56 | -0.11 | -0.28 | 0.67 | -0.01 | 0.86 | -0.03 | -0.86 | 0.34 | 0.70 | 0.32 |
| H57 | -0.11 | -0.02 | -1.19 | -0.44 | -0.68 | -0.32 | 1.78 | 0.08 | -1.49 | -0.69 |
| H58 | 0.05 | 0.01 | 0.06 | 0.02 | -0.43 | -0.19 | 0.41 | 0.02 | -0.64 | 0.13 |
| H59 | -0.33 | -0.06 | -1.26 | -0.47 | -0.15 | -0.17 | 1.04 | 0.05 | -1.33 | -1.07 |
| H60 | 0.03 | 0.00 | -0.58 | -0.21 | -0.54 | -0.46 | 1.28 | 0.06 | -1.61 | -0.13 |
| H61 | -0.11 | -0.02 | -0.59 | -0.22 | -0.31 | -0.06 | 0.86 | 0.04 | -1.31 | -1.16 |
| H62 | -0.07 | -0.01 | -0.71 | -0.26 | -0.20 | -0.42 | 0.89 | 0.04 | -1.31 | -1.55 |
| H63 | 0.09 | 0.12 | 0.22 | 0.30 | 0.71 | 0.09 | -0.17 | -0.54 | -0.12 | 0.16 |
| H64 | 0.12 | 0.02 | 0.63 | 0.23 | -0.31 | -0.07 | 0.01 | 0.00 | 0.34 | -0.65 |
| H65 | 0.14 | 0.02 | 0.40 | 0.05 | 0.30 | -1.58 | -0.20 | 0.04 | 1.02 | -1.12 |
| H66 | 0.01 | 0.00 | 0.06 | 0.02 | -0.08 | 0.38 | 0.12 | 0.01 | -0.02 | -0.37 |
| H67 | -0.20 | 0.32 | 0.21 | -0.10 | 0.21 | -1.08 | 0.01 | -0.41 | 0.52 | -0.21 |
| H68 | 0.41 | -0.67 | 0.27 | -0.13 | 1.02 | 0.53 | -0.43 | -0.30 | 0.89 | -0.11 |
| H69 | 0.15 | -0.25 | 0.00 | 0.00 | 0.00 | -0.23 | 0.02 | -0.14 | 1.01 | 0.63 |
| H70 | -0.01 | 0.01 | 0.04 | -0.02 | 0.41 | 0.17 | -0.65 | -0.69 | 0.90 | -0.40 |
| H71 | -0.19 | 0.32 | 0.16 | -0.07 | 0.11 | -0.62 | -0.33 | -0.65 | 0.05 | -0.45 |
| H72 | -0.11 | 0.18 | 0.18 | -0.08 | 1.12 | 0.12 | -0.39 | -0.61 | 0.65 | -0.40 |
| H73 | -0.01 | 0.01 | 0.03 | -0.01 | 0.50 | -0.22 | -0.45 | -0.42 | 0.67 | 0.40 |
| H74 | 0.01 | -0.01 | -0.05 | 0.02 | 0.78 | -0.14 | -0.29 | -0.09 | 0.66 | -0.25 |
| H75 | -0.23 | 0.37 | -0.01 | 0.00 | 0.42 | -0.28 | -0.29 | -0.23 | 0.58 | -0.93 |
| H76 | 0.14 | -0.23 | 0.29 | -0.14 | 0.49 | 0.15 | -0.23 | -0.11 | 1.13 | 0.42 |
| H77 | 0.23 | -0.37 | 0.11 | -0.05 | 0.20 | 0.23 | -0.41 | -0.46 | 0.63 | 0.29 |
| H78 | -0.43 | 0.53 | -0.50 | 1.20 | 0.65 | 0.40 | -0.10 | 0.13 | -0.41 | -0.34 |
| H79 | -0.36 | 0.10 | -0.33 | 0.09 | 0.27 | 1.16 | 0.15 | 0.56 | -0.45 | -0.76 |
| H80 | -0.49 | 0.08 | -0.30 | 0.27 | -1.41 | 0.37 | 1.09 | 0.30 | -0.06 | -0.16 |
| H81 | -0.91 | 0.15 | -1.15 | -0.31 | -0.95 | -0.06 | 1.30 | 0.49 | -1.19 | 2.58 |
| H82 | -0.64 | -0.56 | -1.47 | 0.26 | -1.48 | -0.09 | 0.90 | 1.85 | -0.20 | 2.03 |
| H83 | 0.31 | -0.53 | 0.56 | -0.61 | -0.77 | 0.80 | -0.05 | 1.28 | 0.38 | 1.43 |
| H84 | -0.40 | -0.14 | 0.00 | -0.42 | -0.61 | 1.74 | -0.80 | 0.61 | 0.79 | 0.91 |
| H85 | -0.32 | 0.31 | -0.69 | -0.85 | -1.31 | 0.21 | -0.30 | -0.26 | 0.84 | 0.61 |
| H86 | -0.47 | -0.46 | -0.38 | 0.25 | -1.87 | 0.26 | 0.92 | 1.25 | 0.18 | 1.53 |
| H87 | 0.23 | 0.04 | 0.73 | 0.27 | -1.18 | 0.04 | -0.33 | -0.01 | -0.22 | 1.78 |
| H88 | -0.04 | 0.01 | -0.48 | -0.16 | -1.44 | -0.10 | 1.56 | 0.10 | -1.19 | 2.26 |
| H89 | -0.26 | 0.04 | -0.13 | -0.05 | 0.07 | -0.18 | -0.81 | 0.04 | 1.55 | 0.66 |
| H90 | -0.01 | 0.00 | 0.09 | 0.04 | -0.74 | 0.03 | -0.34 | -0.01 | -0.52 | 1.59 |
| H91 | -0.39 | 0.04 | -0.24 | 0.04 | -1.25 | 0.53 | 0.48 | -0.90 | -0.98 | 0.74 |
| H92 | -0.69 | -0.07 | -0.74 | 0.26 | -1.17 | -0.26 | 0.95 | 0.11 | -1.03 | 0.78 |
| H93 | -0.17 | -0.15 | -0.50 | 0.15 | -1.13 | 0.11 | -0.13 | 0.07 | -0.24 | -0.02 |
| H94 | -0.30 | 0.03 | -0.57 | 0.09 | -0.37 | 0.10 | 0.20 | -0.36 | 0.39 | 1.61 |
| H95 | -0.18 | 0.10 | -0.42 | 0.16 | -0.67 | -0.27 | 0.16 | -0.45 | -0.16 | 1.33 |
| H96 | -0.15 | 0.06 | -0.40 | 0.07 | -1.30 | 0.22 | 0.16 | -1.36 | -0.80 | 0.58 |
| H97 | -0.46 | 0.09 | -0.14 | 0.31 | -1.36 | 0.68 | 0.14 | -1.40 | 0.03 | 1.94 |
| H98 | -0.44 | 0.06 | -0.68 | -0.23 | -1.33 | -0.36 | 1.26 | 0.04 | -2.06 | 0.68 |
| H99 | -0.85 | -0.03 | -1.36 | 0.23 | -1.07 | -0.83 | 1.30 | 0.18 | -1.02 | 0.70 |
| Variance (%) | 52 | 24 | 73 | 8 | 81 | 10 | 57 | 31 | 53 | 24 |

Supplementary Table S6. Differences between hybrid groups (*P*-values) for yield BLUPs under *Striga* infestation and non-infested conditions at Abuja and Mokwa in Nigeria during the main cropping season across five years

| Groups | DTSTR hybrids | STR commercial hybrids | Non-DTSTR commercial hybrids |
| --- | --- | --- | --- |
|  | *Striga* infested | | |
| DTSTR hybrids |  | 0.718 | <.0001 |
| STR commercial hybrids | 0.718 |  | 0.0005 |
| Non-DTSTR commercial hybrids | <.0001 | 0.0005 |  |
|  | *Striga* non-infested | | |
| DTSTR hybrids |  | 0.7757 | 0.0004 |
| STR commercial hybrids | 0.7757 |  | 0.3381 |
| Non-DTSTR commercial hybrids | 0.0004 | 0.3381 |  |

Supplementary Table S7. Differences between hybrid groups (*P*-values) for yield BLUPs across diverse rainfed field environments (MET) and five years.

| Groups | DTSTR hybrids | STR commercial hybrids | Non-DTSTR commercial hybrids |
| --- | --- | --- | --- |
| DTSTR hybrids |  | 0.7757 | 0.0004 |
| STR commercial hybrids | 0.7757 |  | 0.3381 |
| Non-DTSTR commercial hybrids | 0.0004 | 0.3381 |  |

Supplementary Table S8. Ranges and means of the best linear unbiased predictors for grain yield of selected hybrid groups evaluated under managed drought stress and well watered conditions at Ikenne for five years.

|  |  | Managed drought stress | | | | Full irrigation | | | |
| --- | --- | --- | --- | --- | --- | --- | --- | --- | --- |
| Year | Group | 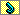Minimum | Maximum | Mean | Standard error | Minimum | Maximum | Mean | Standard error |
| 2012 | DSTST | 1340 | 2204 | 1738 | 115 | 3584 | 5725 | 4857 | 318 |
| 2012 | STR-Com | 845 | 982 | 914 |  | 2100 | 2802 | 2451 |  |
| 2012 | Local | 413 | 413 | 413 |  | 1031 | 1031 | 1031 |  |
| 2013 | DSTST | 2877 | 4115 | 3337 | 156 | 3391 | 4565 | 4020 | 134 |
| 2013 | STR-Com | 2242 | 2424 | 2333 |  | 3278 | 3516 | 3397 |  |
| 2013 | Local | 1238 | 1238 | 1238 |  | 2169 | 2169 | 2169 |  |
| 2014 | DSTST | 1583 | 2453 | 1999 | 79 | 4869 | 5793 | 5368 | 104 |
| 2014 | STR-Com | 1743 | 1925 | 1834 |  | 4794 | 5008 | 4901 |  |
| 2014 | Local | 1179 | 1179 | 1179 |  | 3936 | 3936 | 3936 |  |
| 2015 | DSTST | 711 | 1641 | 1196 | 72 | 3547 | 4689 | 4073 | 179 |
| 2015 | STR-Com | 858 | 1042 | 950 |  | 2448 | 3230 | 2839 |  |
| 2015 | Local | 504 | 504 | 504 |  | 1841 | 1841 | 1841 |  |
| 2016 | DSTST | 741 | 1352 | 1048 | 58 | 3579 | 5194 | 4804 | 131 |
| 2016 | STR-Com | 487 | 776 | 632 |  | 3960 | 4317 | 4139 |  |
| 2016 | Local | 667 | 667 | 667 |  | 3443 | 3443 | 3443 |  |

Supplementary Table S9. Average grain yield differences among hybrid groups (DTSTR (1), STR(2) and Local(3)) tested with The Least Squares Means/PDIFF (LSMEANS/PDIFF) option in SAS (2016).

| Managed drought stress (MDS) | | |  | Full Irrigation (WW) | | | |
| --- | --- | --- | --- | --- | --- | --- | --- |
| 2012 | | | | 2012 | | | |
| Least Squares Means for effect GROUP | | | | Least Squares Means for effect GROUP | | | |
| Pr > |t| for H0: LSMean(i)=LSMean(j) | | | | Pr > |t| for H0: LSMean(i)=LSMean(j) | | | |
| Group | 1 | 2 | 3 | Group | 1 | 2 | 3 |
| 1 |  | 0.0011 | 0.0003 | 1 |  | 0.0002 | <.0001 |
| 2 | 0.0011 |  | 0.1487 | 2 | 0.0002 |  | 0.0909 |
| 3 | 0.0003 | 0.1487 |  | 3 | <.0001 | 0.0909 |  |
|  |  |  |  |  |  |  |  |
| 2013 | | | | 2013 | | | |
| Least Squares Means for effect GROUP | | | | Least Squares Means for effect GROUP | | | |
| Pr > |t| for H0: LSMean(i)=LSMean(j) | | | | Pr > |t| for H0: LSMean(i)=LSMean(j) | | | |
| Group | 1 | 2 | 3 | Group | 1 | 2 | 3 |
| 1 |  | 0.0005 | <.0001 | 1 |  | 0.0165 | <.0001 |
| 2 | 0.0005 |  | 0.0084 | 2 | 0.0165 |  | 0.0051 |
| 3 | <.0001 | 0.0084 |  | 3 | <.0001 | 0.0051 |  |
|  |  |  |  |  |  |  |  |
| 2014 | | | | 2014 | | | |
| Least Squares Means for effect GROUP | | | | Least Squares Means for effect GROUP | | | |
| Pr > |t| for H0: LSMean(i)=LSMean(j) | | | | Pr > |t| for H0: LSMean(i)=LSMean(j) | | | |
| Group | 1 | 2 | 3 | Group | 1 | 2 | 3 |
| 1 |  | 0.4401 | 0.012 | 1 |  | 0.0211 | <.0001 |
| 2 | 0.4401 |  | 0.0719 | 2 | 0.0211 |  | 0.0052 |
| 3 | 0.012 | 0.0719 |  | 3 | <.0001 | 0.0052 |  |
|  |  |  |  |  |  |  |  |
| 2015 | | | | 2015 | | | |
| Least Squares Means for effect GROUP | | | | Least Squares Means for effect GROUP | | | |
| Pr > |t| for H0: LSMean(i)=LSMean(j) | | | | Pr > |t| for H0: LSMean(i)=LSMean(j) | | | |
| Group | 1 | 2 | 3 | Group | 1 | 2 | 3 |
| 1 |  | 0.2204 | 0.0195 | 1 |  | 0.0007 | <.0001 |
| 2 | 0.2204 |  | 0.1725 | 2 | 0.0007 |  | 0.0491 |
| 3 | 0.0195 | 0.1725 |  | 3 | <.0001 | 0.0491 |  |
|  |  |  |  |  |  |  |  |
| 2016 | | | | 2016 | | | |
| Least Squares Means for effect GROUP | | | | Least Squares Means for effect GROUP | | | |
| Pr > |t| for H0: LSMean(i)=LSMean(j) | | | | Pr > |t| for H0: LSMean(i)=LSMean(j) | | | |
| Group | 1 | 2 | 3 | Group | 1 | 2 | 3 |
| 1 |  | 0.0125 | 0.0763 | 1 |  | 0.0489 | 0.0062 |
| 2 | 0.0125 |  | 0.8823 | 2 | 0.0489 |  | 0.1859 |
| 3 | 0.0763 | 0.8823 |  | 3 | 0.0062 | 0.1859 |  |

Supplementary Table S10. Ranges and means of the best linear unbiased predictors for grain yield of selected hybrid groups evaluated under *Striga* infested and non-infested conditions at two locations for five years.

|  |  |  | *Striga* infested | | | | *Striga* non-infested | | | |
| --- | --- | --- | --- | --- | --- | --- | --- | --- | --- | --- |
| Year | Location | Geoup | Minimum | Maximum | Mean | Standard Error | Minimum | Maximum | Mean | Standard Error |
| 2012 | Kubwa | DSTST | 3854 | 5414 | 4841 | 118 | 4830 | 5920 | 5505 | 84 |
| 2012 | Kubwa | STR-Com | 4045 | 4686 | 4366 |  | 5290 | 5480 | 5385 |  |
| 2012 | Kubwa | Local | 3588 | 3588 | 3588 |  | 4820 | 4820 | 4820 |  |
| 2012 | Mokwa | DSTST | 2305 | 3984 | 29356 | 118 | 2400 | 3060 | 2816 | 52 |
| 2012 | Mokwa | STR-Com | 2667 | 2699 | 2683 |  | 2680 | 2790 | 2735 |  |
| 2012 | Mokwa | Local | 1970 | 1970 | 1970 |  | 2370 | 2370 | 2370 |  |
| 2013 | Kubwa | DSTST | 4026 | 5092 | 4489 | 140 | 4320 | 5490 | 4904 | 124 |
| 2013 | Kubwa | STR-Com | 3971 | 4085 | 4028 |  | 4240 | 4280 | 4260 |  |
| 2013 | Kubwa | Local | 2347 | 2347 | 2347 |  | 3300 | 3300 | 3300 |  |
| 2013 | Mokwa | DSTST | 3443 | 4607 | 4064 | 155 | 3800 | 4900 | 4477 | 136 |
| 2013 | Mokwa | STR-Com | 4057 | 4097 | 4077 |  | 3810 | 4100 | 3955 |  |
| 2013 | Mokwa | Local | 1709 | 1709 | 1709 |  | 2580 | 2580 | 2580 |  |
| 2014 | Kubwa | DSTST | 3291 | 4603 | 4080 | 200 | 4460 | 5610 | 4966 | 133 |
| 2014 | Kubwa | STR-Com | 3536 | 3961 | 3749 |  | 4220 | 4470 | 4345 |  |
| 2014 | Kubwa | Local | 957 | 957 | 957 |  | 3190 | 3190 | 3190 |  |
| 2014 | Mokwa | DSTST | 3451 | 4120 | 3808 | 102 | 3420 | 5790 | 4554 | 186 |
| 2014 | Mokwa | STR-Com | 3739 | 3769 | 3754 |  | 3750 | 3890 | 3820 |  |
| 2014 | Mokwa | Local | 2214 | 2214 | 2214 |  | 2630 | 2630 | 2630 |  |
| 2015 | Kubwa | DSTST | 2972 | 5464 | 4584 | 181 | 5480 | 6410 | 5966 | 109 |
| 2015 | Kubwa | STR-Com | 3520 | 4481 | 4001 |  | 5430 | 5600 | 5515 |  |
| 2015 | Kubwa | Local | 2803 | 2803 | 2803 |  | 4590 | 4590 | 4590 |  |
| 2015 | Mokwa | DSTST | 2956 | 3993 | 3610 | 107 | 3290 | 4050 | 3738 | 73 |
| 2015 | Mokwa | STR-Com | 3114 | 3509 | 3312 |  | 3440 | 3460 | 3450 |  |
| 2015 | Mokwa | Local | 2060 | 2060 | 2060 |  | 2790 | 2790 | 2790 |  |
| 2016 | Kubwa | DSTST | 4110 | 5470 | 4784 | 222 | 4390 | 6650 | 5322 | 162 |
| 2016 | Kubwa | STR-Com | 4443 | 4825 | 4634 |  | 4680 | 5090 | 4885 |  |
| 2016 | Kubwa | Local | 1346 | 1346 | 1346 |  | 3870 | 3870 | 3870 |  |
| 2016 | Mokwa | DSTST | 1356 | 2537 | 2099 | 86 | 1980 | 3790 | 2723 | 132 |
| 2016 | Mokwa | STR-Com | 1459 | 1989 | 1724 |  | 1940 | 2430 | 2185 |  |
| 2016 | Mokwa | Local | 1356 | 1356 | 1356 |  | 1580 | 1580 | 1580 |  |

Supplementary Table S11. Average grain yield differences among hybrid groups (DTSTR (1), STR(2) and Local(3)) tested with The Least Squares Means/PDIFF (LSMEANS/PDIFF) option in SAS (2016).

| *Striga* infestation (STRIN) | | | | *Striga* non-infested (STRIN) | | | |
| --- | --- | --- | --- | --- | --- | --- | --- |
| ENV1 | | | | ENV1 | | | |
| Least Squares Means for effect GROUP | | | | Least Squares Means for effect GROUP | | | |
| Pr > |t| for H0: LSMean(i)=LSMean(j) | | | | Pr > |t| for H0: LSMean(i)=LSMean(j) | | | |
| Group | 1 | 2 | 3 | Group | 1 | 2 | 3 |
| 1 |  | 0.123 | 0.007 | 1 |  | 0.6307 | 0.0597 |
| 2 | 0.123 |  | 0.1201 | 2 | 0.6307 |  | 0.1752 |
| 3 | 0.007 | 0.1201 |  | 3 | 0.0597 | 0.1752 |  |
|  |  |  |  |  |  |  |  |
| ENV2 | | | | ENV2 | | | |
| Least Squares Means for effect GROUP | | | | Least Squares Means for effect GROUP | | | |
| Pr > |t| for H0: LSMean(i)=LSMean(j) | | | | Pr > |t| for H0: LSMean(i)=LSMean(j) | | | |
| Group | 1 | 2 | 3 | Group | 1 | 2 | 3 |
| 1 |  | 0.4573 | 0.0511 | 1 |  | 0.5945 | 0.0454 |
| 2 | 0.4573 |  | 0.2044 | 2 | 0.5945 |  | 0.1506 |
| 3 | 0.0511 | 0.2044 |  | 3 | 0.0454 | 0.1506 |  |
|  |  |  |  |  |  |  |  |
| ENV3 | | | | ENV3 | | | |
| Least Squares Means for effect GROUP | | | | Least Squares Means for effect GROUP | | | |
| Pr > |t| for H0: LSMean(i)=LSMean(j) | | | | Pr > |t| for H0: LSMean(i)=LSMean(j) | | | |
| Group | 1 | 2 | 3 | Group | 1 | 2 | 3 |
| 1 |  | 0.0274 | <.0001 | 1 |  | 0.0142 | 0.0002 |
| 2 | 0.0274 |  | <.0001 | 2 | 0.0142 |  | 0.022 |
| 3 | <.0001 | <.0001 |  | 3 | 0.0002 | 0.022 |  |
|  |  |  |  |  |  |  |  |
| ENV4 | | | | ENV4 | | | |
| Least Squares Means for effect GROUP | | | | Least Squares Means for effect GROUP | | | |
| Pr > |t| for H0: LSMean(i)=LSMean(j) | | | | Pr > |t| for H0: LSMean(i)=LSMean(j) | | | |
| Group | 1 | 2 | 3 | Group | 1 | 2 | 3 |
| 1 |  | 0.9557 | <.0001 | 1 |  | 0.0474 | <.0001 |
| 2 | 0.9557 |  | <.0001 | 2 | 0.0474 |  | 0.0033 |
| 3 | <.0001 | <.0001 |  | 3 | <.0001 | 0.0033 |  |
|  |  |  |  |  |  |  |  |
| ENV5 | | | | ENV5 | | | |
| Least Squares Means for effect GROUP | | | | Least Squares Means for effect GROUP | | | |
| Pr > |t| for H0: LSMean(i)=LSMean(j) | | | | Pr > |t| for H0: LSMean(i)=LSMean(j) | | | |
| Group | 1 | 2 | 3 | Group | 1 | 2 | 3 |
| 1 |  | 0.2364 | <.0001 | 1 |  | 0.0228 | 0.0001 |
| 2 | 0.2364 |  | <.0001 | 2 | 0.0228 |  | 0.0109 |
| 3 | <.0001 | <.0001 |  | 3 | 0.0001 | 0.0109 |  |
|  |  |  |  |  |  |  |  |
| ENV6 | | | | ENV6 | | | |
| Least Squares Means for effect GROUP | | | | Least Squares Means for effect GROUP | | | |
| Pr > |t| for H0: LSMean(i)=LSMean(j) | | | | Pr > |t| for H0: LSMean(i)=LSMean(j) | | | |
| Group | 1 | 2 | 3 | Group | 1 | 2 | 3 |
| 1 |  | 0.6997 | <.0001 | 1 |  | 0.1368 | 0.0091 |
| 2 | 0.6997 |  | <.0001 | 2 | 0.1368 |  | 0.1363 |
| 3 | <.0001 | <.0001 |  | 3 | 0.0091 | 0.1363 |  |
|  |  |  |  |  |  |  |  |
| ENV7 | | | | ENV7 | | | |
| Least Squares Means for effect GROUP | | | | Least Squares Means for effect GROUP | | | |
| Pr > |t| for H0: LSMean(i)=LSMean(j) | | | | Pr > |t| for H0: LSMean(i)=LSMean(j) | | | |
| Group | 1 | 2 | 3 | Group | 1 | 2 | 3 |
| 1 |  | 0.2402 | 0.0161 | 1 |  | 0.0654 | 0.0005 |
| 2 | 0.2402 |  | 0.1425 | 2 | 0.0654 |  | 0.0239 |
| 3 | 0.0161 | 0.1425 |  | 3 | 0.0005 | 0.0239 |  |
|  |  |  |  |  |  |  |  |
| ENV8 | | | | ENV8 | | | |
| Least Squares Means for effect GROUP | | | | Least Squares Means for effect GROUP | | | |
| Pr > |t| for H0: LSMean(i)=LSMean(j) | | | | Pr > |t| for H0: LSMean(i)=LSMean(j) | | | |
| Group | 1 | 2 | 3 | Group | 1 | 2 | 3 |
| 1 |  | 0.1273 | <.0001 | 1 |  | 0.0657 | 0.0003 |
| 2 | 0.1273 |  | 0.0009 | 2 | 0.0657 |  | 0.0135 |
| 3 | <.0001 | 0.0009 |  | 3 | 0.0003 | 0.0135 |  |
|  |  |  |  |  |  |  |  |
| ENV9 | | | | ENV9 | | | |
| Least Squares Means for effect GROUP | | | | Least Squares Means for effect GROUP | | | |
| Pr > |t| for H0: LSMean(i)=LSMean(j) | | | | Pr > |t| for H0: LSMean(i)=LSMean(j) | | | |
| Group | 1 | 2 | 3 | Group | 1 | 2 | 3 |
| 1 |  | 0.6396 | <.0001 |  |  | 0.347 | 0.0333 |
| 2 | 0.6396 |  | <.0001 | 1 | 0.347 |  | 0.1848 |
| 3 | <.0001 | <.0001 |  | 2 | 0.0333 | 0.1848 |  |
|  |  |  |  | 3 |  |  |  |
| ENV10 | | | | ENV10 | | | |
| Least Squares Means for effect GROUP | | | | Least Squares Means for effect GROUP | | | |
| Pr > |t| for H0: LSMean(i)=LSMean(j) | | | | Pr > |t| for H0: LSMean(i)=LSMean(j) | | | |
| Group | 1 | 2 | 3 | Group | 1 | 2 | 3 |
| 1 |  | 0.1238 | 0.0328 | 1 |  | 0.1558 | 0.0354 |
| 2 | 0.1238 |  | 0.3386 | 2 | 0.1558 |  | 0.3153 |
| 3 | 0.0328 | 0.3386 |  | 3 | 0.0354 | 0.3153 |  |

Supplementary Table S12. Ranges and means of the best linear unbiased predictors for grain yield of selected hybrid groups evaluated in multiple field environments for five years.

| Year | Geoup | 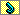Minimum | Maximum | Mean | Standard Error |
| --- | --- | --- | --- | --- | --- |
| 2012 | DSTST | 3013 | 3406 | 3175 | 65 |
| 2012 | STR-Com | 2823 | 2979 | 2901 |
| 2012 | Local | 2476 | 2476 | 2476 |
| 2013 | DSTST | 4073 | 4590 | 4310 | 71 |
| 2013 | STR-Com | 3808 | 3974 | 3891 |
| 2013 | Local | 3495 | 3495 | 3495 |
| 2014 | DSTST | 3700 | 4245 | 3965 | 90 |
| 2014 | STR-Com | 3163 | 3460 | 3312 |
| 2014 | Local | 3275 | 3275 | 3275 |
| 2015 | DSTST | 3594 | 4133 | 3877 | 82 |
| 2015 | STR-Com | 2959 | 3104 | 3032 |
| 2015 | Local | 2945 | 2945 | 2945 |
| 2016 | DSTST | 3221 | 3921 | 3717 | 50 |
| 2016 | STR-Com | 3039 | 3185 | 3112 |
| 2016 | Local | 2722 | 2722 | 2722 |

Supplementary Table S13. Average grain yield differences among hybrid groups (DTSTR (1), STR(2) and Local(3)) tested with The Least Squares Means/PDIFF (LSMEANS/PDIFF) option in SAS (2016).

| Multi-environment trials | | | | |
| --- | --- | --- | --- | --- |
| 2012 | | | | |
| Least Squares Means for effect GROUP | | | | |
| Pr > |t| for H0: LSMean(i)=LSMean(j) | | | | |
| Group | 1 | | 2 | 3 |
| 1 |  | | 0.0016 | <.0001 |
| 2 | 0.0016 | |  | 0.0022 |
| 3 | <.0001 | | 0.0022 |  |
|  |  | |  |  |
| 2013 | | | | |
| Least Squares Means for effect GROUP | | | | |
| Pr > |t| for H0: LSMean(i)=LSMean(j) | | | | |
| Group | 1 | | 2 | 3 |
| 1 |  | | 0.0019 | <.0001 |
| 2 | 0.0019 | |  | 0.0429 |
| 3 | <.0001 | | 0.0429 |  |
|  |  | |  |  |
| 2014 | | | | |
| Least Squares Means for effect GROUP | | | | |
| Pr > |t| for H0: LSMean(i)=LSMean(j) | | | | |
| Group | 1 | | 2 | 3 |
| 1 |  | | <.0001 | 0.0004 |
| 2 | <.0001 | |  | 0.8371 |
| 3 | 0.0004 | | 0.8371 |  |
|  |  | |  |  |
| 2015 | | | | |
| Least Squares Means for effect GROUP | | | | |
| Pr > |t| for H0: LSMean(i)=LSMean(j) | | | | |
| Group | 1 | | 2 | 3 |
| 1 |  | | <.0001 | <.0001 |
| 2 | <.0001 | |  | 0.6383 |
| 3 | <.0001 | | 0.6383 |  |
|  |  |  | |  |
| 2016 | | | | |
| Least Squares Means for effect GROUP | | | | |
| Pr > |t| for H0: LSMean(i)=LSMean(j) | | | | |
| Group | 1 | 2 | | 3 |
| 1 |  | 0.0003 | | <.0001 |
| 2 | 0.0003 |  | | 0.0784 |
| 3 | <.0001 | 0.0784 | |  |
